# Antigen presentation deficiency, mesenchymal differentiation, and resistance to immunotherapy in the murine syngeneic CT2A tumor model

**DOI:** 10.3389/fimmu.2023.1297932

**Published:** 2023-12-28

**Authors:** J. Bryan Iorgulescu, Neil Ruthen, Ryuhjin Ahn, Eleni Panagioti, Prafulla C. Gokhale, Martha Neagu, Maria C. Speranza, Benjamin K. Eschle, Kara M. Soroko, Raziye Piranlioglu, Meenal Datta, Shanmugarajan Krishnan, Kathleen B. Yates, Gregory J. Baker, Rakesh K. Jain, Mario L. Suvà, Donna Neuberg, Forest M. White, E. Antonio Chiocca, Gordon J. Freeman, Arlene H. Sharpe, Catherine J. Wu, David A. Reardon

**Affiliations:** ^1^ Department of Medical Oncology, Dana-Farber Cancer Institute, Boston, MA, United States; ^2^ Department of Pathology, Brigham and Women’s Hospital, Harvard Medical School, Boston, MA, United States; ^3^ The Eli and Edythe L. Broad Institute of MIT and Harvard, Cambridge, MA, United States; ^4^ Department of Biological Engineering, Massachusetts Institute of Technology, Cambridge, MA, United States; ^5^ Koch Institute for Integrative Cancer Research, Massachusetts Institute of Technology, Cambridge, MA, United States; ^6^ Department of Neurosurgery, Brigham and Women’s Hospital and Harvard Medical School, Boston, MA, United States; ^7^ Experimental Therapeutics Core and Belfer Center for Applied Cancer Science, Dana-Farber Cancer Institute, Boston, MA, United States; ^8^ Department of Immunology, Blavatnik Institute, Harvard Medical School, Boston, MA, United States; ^9^ Edwin L. Steele Laboratories for Tumor Biology, Massachusetts General Hospital, Harvard Medical School, Boston, MA, United States; ^10^ Department of Aerospace and Mechanical Engineering, University of Notre Dame, Notre Dame, IN, United States; ^11^ Center for Cancer Research, Massachusetts General Hospital, Boston, MA, United States; ^12^ Laboratory of Systems Pharmacology, Program in Therapeutic Science, Harvard Medical School, Boston, MA, United States; ^13^ Ludwig Center for Cancer Research at Harvard, Harvard Medical School, Boston, MA, United States; ^14^ Department of Pathology, Massachusetts General Hospital, Harvard Medical School, Boston, MA, United States

**Keywords:** immunotherapy, resistance, mouse model, cancer, antigen presentation machinery, glioblastoma, mesenchymal

## Abstract

**Background:**

The GL261 and CT2A syngeneic tumor lines are frequently used as immunocompetent orthotopic mouse models of human glioblastoma (huGBM) but demonstrate distinct differences in their responses to immunotherapy.

**Methods:**

To decipher the cell-intrinsic mechanisms that drive immunotherapy resistance in CT2A-luc and to define the aspects of human cancer biology that these lines can best model, we systematically compared their characteristics using whole exome and transcriptome sequencing, and protein analysis through immunohistochemistry, Western blot, flow cytometry, immunopeptidomics, and phosphopeptidomics.

**Results:**

The transcriptional profiles of GL261-luc2 and CT2A-luc tumors resembled those of some huGBMs, despite neither line sharing the essential genetic or histologic features of huGBM. Both models exhibited striking hypermutation, with clonal hotspot mutations in RAS genes (*Kras* p.G12C in GL261-luc2 and *Nras* p.Q61L in CT2A-luc). CT2A-luc distinctly displayed mesenchymal differentiation, upregulated angiogenesis, and multiple defects in antigen presentation machinery (e.g. *Tap1* p.Y488C and *Psmb8* p.A275P mutations) and interferon response pathways (e.g. copy number losses of loci including IFN genes and reduced phosphorylation of JAK/STAT pathway members). The defect in MHC class I expression could be overcome in CT2A-luc by interferon-γ treatment, which may underlie the modest efficacy of some immunotherapy combinations. Additionally, CT2A-luc demonstrated substantial baseline secretion of the CCL-2, CCL-5, and CCL-22 chemokines, which play important roles as myeloid chemoattractants.

**Conclusion:**

Although the clinical contexts that can be modeled by GL261 and CT2A for huGBM are limited, CT2A may be an informative model of immunotherapy resistance due to its deficits in antigen presentation machinery and interferon response pathways.

## Introduction

Therapeutic blockade of inhibitory immune checkpoint pathways (*e.g.*, PD-1/PD-L1 and CTLA-4) has transformed the care of patients across multiple cancer types, including many formerly intractable advanced cancers (1). However, for human IDH-wildtype glioblastoma (huGBM)—a common and aggressive brain cancer typified by a median survival of just 14-22 months depending on *MGMT* promoter methylation status (2, 3)—the near-uniform negative results of single-agent immune checkpoint blockade (ICB) clinical trials have been disappointing (4–6). The intracranial location of huGBM and the blood brain barrier by themselves do not appear to preclude effective anti-tumoral immunity, since ICB has been successful in treating brain metastases from a variety of primary cancers (7, 8). In contrast to many brain metastasis types, however, huGBM is characterized by a ‘cold’ (i.e., a paucity of T cells) immune microenvironment. This immuno-resistance has been also ascribed to other multifaceted sources, including: 1) a dominance of suppressive myeloid cells; 2) a low tumor mutational burden; 3) limited PD-L1 expression; 4) T cell sequestration in the bone marrow; and 5) immunosuppression mediated by the frequent need for high-dose corticosteroids to treat symptomatic cerebral edema (9–12). In an effort to overcome these barriers, numerous immunotherapeutic approaches are currently under clinical investigation for huGBM, including immunomodulatory agents (e.g. NCT04547777), peptide vaccination (e.g. NCT02287428), oncolytic virotherapy (e.g. NCT03152318), adoptive cell therapy (e.g. NCT05660369), and next-generation immune checkpoint modulators (e.g. NCT04826393) – which have been extensively reviewed elsewhere (10).

A valuable cornerstone of preclinical oncology research is the use of syngeneic orthotopic murine cancer lines as immunocompetent models of cancer. For huGBM, the two frequently used lines are GL261 and CT2A, both of which were generated by injecting the carcinogen methylcholanthrene into the brains of mice (13–15). As with other methylcholanthrene-derived cancers, both GL261 and CT2A exhibit a striking degree of hypermutation, which contrasts with the low tumor mutational burden typically observed in huGBM (16, 17). However, whereas GL261 is readily responsive to several immunotherapies, we and others have previously demonstrated the broad resistance of the syngeneic CT2A mouse model to diverse single-agent immunotherapies, including PD-1/PD-L1 pathway inhibition, vaccine therapy, and oncolytic virotherapy (12, 17–21) – a phenotype that is observed even in CT2A lines that express the immunogenic luciferase protein (12, 19). Given the urgent need to devise better therapeutics for huGBM, our specific aim was to decipher the cell-intrinsic mechanisms driving the broad immuno-resistance in CT2A and to define the aspects of human cancer biology that these lines can best model for further preclinical research. To achieve this aim, we systematically characterized these lines using multi-modal profiling, including genomic, transcriptomic, protein, immuno-peptidomic, and phospho-peptidomic analyses.

## Materials and methods

All animal experiments were approved by the Dana-Farber Cancer Institute and Harvard Medical School Animal Care and Use Committees.

### Cell culture

Luciferase-transduced GL261 cells (GL261-luc2; RRID: CVCL_X986) were obtained from PerkinElmer (Waltham, MA). CT2A cells were obtained from Thomas Seyfried (Boston College; RRID: CVCL_ZJ44) and transduced using firefly luciferase lentiviral particles (CT2A-luc; Kerafast Inc., Boston, MA). Cell lines were expanded and frozen at the same generation. For experiments, cells were thawed and cultured at 37°C in a humidified incubator with 5% CO_2_ using Dulbecco’s Modified Eagle Medium supplemented with 10% heat-inactivated fetal calf serum and 100 μg/mL G418 (for GL261-luc2) or 2 μg/mL puromycin (for CT2A-luc). Cultures were regularly tested as negative for mycoplasma. Luciferase was used to enable bioluminescent imaging and ensure that tumors’ engraftments were comparable prior to extraction or therapeutic experiments.

For the second set of experiments (Cohort B), a truncated human *CD19* reporter gene was introduced into GL261-luc2 and CT2A-luc as previously described (22). The purity of hCD19-positive tumor cells was confirmed by flow cytometry following sorting, using isotype controls for comparison. Unless otherwise noted, all cell lines were grown as adherent cultures. Neurospheres were cultured as previously described (23). All cell lines were fingerprinted using their DNA/RNA sequencing data.

### Intracranial tumor cell inoculation

Thawed cells were cultured for up to three passages prior to intracranial implantation. 1×10^5^ GL261-luc2 cells or 0.25×10^5^ CT2A-luc cells were suspended in phosphate-buffered saline (PBS) and stereotactically injected into the right striatum of anesthetized, female 7-10 week-old, albino C57BL/6 mice (Jackson Laboratory; Bar Harbor, ME). For GL261-hCD19-luc2 and CT2A-hCD19-luc, 2×10^5^ cells were implanted.

### Survival experiments

For checkpoint immunotherapy, all antibodies were injected intraperitoneally as previously described (12, 18). The 332.8H3 mouse anti-mouse PD-1 monoclonal antibody (IgG1; generated in Gordon Freeman’s laboratory; with MOPC21 isotype control [BioXCell, West Lebanon, NH]) and/or CTLA-4 antibody (clone 9D9, BioXCell) were administered as a loading dose (500 μg) on day 6 after tumor implantation, followed by 250 μg injections every 3 days for 7 additional doses. The OX40 antibody (clone OX-86, BioXCell; with rat IgG1 clone HRPN isotype control) was administered as 100 µg weekly, either for 2 doses starting on day 6 for GL261-luc2 or 3 doses starting on day 3 for CT2A-luc. All monoclonal antibodies contained <2 EU/mg endotoxin. Bioluminescent imaging (BLI) was used to identify mice with growing tumor burden for randomization into experimental cohorts, which included 8 mice per experimental arm. Bioluminescence imaging involved subcutaneous injection of D-luciferin and imaging with the IVIS imaging system approximately once each week across experiments, as previously described (18). Mice were euthanized for signs of morbidity or after ≥100 days if healthy appearing.

### Bulk whole exome sequencing (WES) and RNA sequencing (RNAseq)

*In vitro* GL261-luc2 and CT2A-luc cells, and GL261-luc2 and CT2A-luc *ex vivo* bulk tumors (harvested 22-24 days after implantation) were prepared for DNA and RNA extractions, library preparations, and sequencing that were performed at GENEWIZ (South Plainfield, NJ). For *in vitro* GL261-luc2 and CT2A-luc cells, and GL261-luc2 and CT2A-luc *ex vivo* bulk tumors, DNA was extracted using the PureLink Genomic DNA Mini Kit (Thermo Fisher Scientific, Waltham, MA) and the sequencing libraries were prepared using the SureSelectXT Mouse All Exon Kit (Agilent, Santa Clara, CA). Fragmented DNAs were cleaned up, end repaired, and adenylated at the 3’ends. Adapters were ligated to the DNA fragments, which were then enriched with limited cycle PCR. 200 ng adapter-ligated DNA fragments were hybridized with biotinylated RNA baits at 65°C for 24 hours. The hybrid DNAs were captured by streptavidin-coated magnetic beads, extensively washed, and then amplified and indexed with Illumina indexing primers.

Total RNA was extracted using RNeasy Plus Mini kit (Qiagen, Hilden, Germany) and sequencing libraries were prepared using the NEBNext Ultra II RNA Library Prep Kit for Illumina (New England Biolabs, Ipswich, MA). mRNAs were enriched with Oligod(T) beads and fragmented for 15 minutes at 94°C. First-strand and second-strand cDNA were synthesized, end repaired, adenylated at 3’ends, and ligated to universal adapters; followed by index addition and library enrichment by PCR with limited cycles.

For GL261-hCD19-luc2 and CT2A-hCD19-luc, tumor cells were first isolated from dissociated *ex vivo* tumors by bead-based positive magnetic selection for hCD19 (Miltenyi). RNA was isolated using the RNeasy Mini kit. First-strand Illumina-barcoded libraries were generated using the NEB RNA Ultra Directional kit, including 12 cycles of PCR enrichment. Libraries were subsequently sequenced on an Illumina NextSeq500 instrument using paired-end 37 bp reads. The DNA and RNA sequencing libraries were validated on the TapeStation (Agilent Technologies, Palo Alto, CA), multiplexed, and clustered onto flow cells for sequencing using a 2x150 bp Paired End configuration on the Illumina HiSeq. Raw sequence data were converted into fastq files and de-multiplexed using Illumina bcl2fastq 2.17 software. One mis-match was allowed for index sequence identification.

### WES and RNAseq analysis

Default settings were used for all WES and bulk RNAseq analysis tools. For all samples, WES data were aligned to the mm10 reference genome using bwaMem (v0.7.15), then deduplicated and recalibrated with the Picard tools MarkDuplicates, BaseRecalibrator, and ApplyBQSR packaged in GATK (v4.1.8.1). SNVs were identified by consensus calling with Mutect2 (packaged in GATK 4.1.8.1) and Strelka2 (v2.9.3). InDels were identified by consensus calling with Mutect2 and Manta (v1.5.0) packaged with Strelka2. Variants were annotated using Ensembl VEP (v102). Contiguous copy ratio segments were identified with CollectReadCounts, DenoiseReadCounts, and ModelSegments packaged in GATK (v4.1.8.1). Recurrent CNVs were subsequently identified from these segmentation files with GISTIC2.0. All calls were tested against a panel of normals comprising three C57BL/6 tail samples. Tumor mutational burden was estimated based on the SureSelectXT Mouse All Exon Kit’s 49.6 MB capture.

Transcript abundances were estimated from RNA sequencing data directly using Salmon (v1.4.0) with the mm10 reference transcriptome. Differential expression analysis was performed using DESeq2 (v1.30.1), including genes with normalized transcript counts >10. Gene set enrichment analysis with the fgsea R package (v1.20.0) was performed across all genes pre-ranked by log_10_(p-value) * -(sign of the LFC). Additionally, the sequencing data were aligned to the mm10 reference transcriptome with STAR (v2.7.7a) and expression metrics for each gene were computed using RSEM (v1.3.3). Neoantigens were predicted from the aggregated results of the WES and bulk RNAseq analyses using the pVACseq pipeline (pVACtools v2.0.2) and vatools (v4.1.0). MHC I binding predictions were performed using a consensus of NetMHCpan, NetMHC, and PickPocket algorithms. RNA expression between samples was visualized using a heatmap, in which DESeq2 normalization was applied to the matrix of transcript counts, bounded to 1 if normalized value > 1 and to -1 if normalized value < -1, and then scaled to a range of 0 to 1 (by adding 1 to all counts and dividing by 2). Immune cell abundances were estimated from the RNA sequencing of bulk tumors using the murine Microenvironment Cell Population (mMCP) tool, according to default settings (24).

### Immunoprecipitation of MHC class I-bound peptides

Peptide-bound MHC and phosphopeptide samples were analyzed as previously described (25). GL261-luc2 and CT2A-luc tumors were flash-frozen 22-24 days after implantation. Following homogenization and clearing by centrifugation, 1.5 mg of lysate per sample was immunoprecipitated overnight at 4°C with 0.1 mg of anti-H2-K^b^ (clone Y3, BioXCell) and 0.1 mg of anti-H2-D^b^ (clone 28-14-8S; hybridoma from ATCC) bound to 20 μL FastFlow Protein A sepharose beads (GE Healthcare). Beads were washed with TBS and water and then peptide-bound MHCs were eluted with 10% acetic acid. Peptides were separated from antibody and MHC via 10K molecular weight cut-off filters (PALL life sciences), lyophilized, and stored in -80°C before labeling. For multiplexing, lyophilized peptide-bound MHCs were resuspended in 33 μL of labeling buffer (50% ethanol, 150 mM TEAB) and mixed with 40 μg of pre-aliquoted TMTpro 16plex Label Reagent (Thermo Fisher Scientific) resuspended in 10 μL of anhydrous acetonitrile. Labeling reaction occurred on a shaker for 4.5 hours at room temperature and quenched with 0.3% hydroxylamine. Samples were pooled and dried in SpeedVac centrifuge prior to cleaning up with SP3 protocol as previously described (25).

### Phosphopeptide enrichment

Tandem mass tag (TMT)-labeled samples were resuspended in IP buffer (1% Nonidet P-40, 100 mM Tris-HCl, pH 7.4) with protein G agarose beads conjugated to 24 μg of 4G10 V312 IgG and 6 μg PT-66 (Sigma) overnight at 4°C. Beads were washed with 100 mM Tris-HCl (pH 7.4), and eluted twice with 0.2% trifluoroacetic acid for 10 minute at room temperature followed by the enrichment of phosphopeptides using High-Select Fe-NTA enrichment kit (Pierce) with modification to the elution step (20 μL of elution buffer into a 1.7 mL microcentrifuge tube). Eluates were dried and resuspended in 10 μL of 3% acetonitrile in 0.1% formic acid for direct loading onto an in-house packed analytical capillary column (50 μm ID x 10 cm x 5 μm C18 beads; YMC gel). Supernatant from pTyr enrichment was used for fractionation as previously described into 10 fractions using high pH reverse‐phase chromatography on a ZORBAX C18 column. One tenth of each fraction was used for global proteomics analysis and the rest subjected to phosphopeptide enrichment using Fe-NTA enrichment kit for global phosphoproteomic analysis (25).

### Liquid chromatography tandem mass spectrometry

Peptide-bound MHC samples were analyzed using an Exploris 480 Hybrid Quadrupole-Orbitrap mass spectrometer (Thermo Fisher Scientific) coupled to an Agilent 1260 LC system. TMT-labeled peptides were resuspended in 3% acetonitrile/0.1% formic acid and loaded on a precolumn (100 um ID x 10 cm packed in-house with 10 μm C18 beads; YMC gel) connected in tandem to an in-house packed analytical column (50 μm ID × 15 cm and 1.9 μM C18 beads, ReproSil-Pur). Peptides were eluted using a gradient with 70% acetonitrile in 0.2 M acetic acid at the flow rate of 0.2 mL/min and a pre-column split of 2000:1. Standard mass spectrometry parameters were: spray voltage, 2.0 kV, no sheath or auxiliary gas flow, and heated capillary temperature of 275°C. The Exploris was operated in data dependent acquisition mode with the following MS1 parameters: scan range of 350-1200 m/z; resolution of 60,000; normalized AGC target of 300%; automatic IT; and dynamic exclusion (exclude precursors from selection for 30 seconds once fragmented twice within 20 second). Collection of MS2 spectra was performed under the following parameters: 60,000 resolution; isolation width of 0.4 m/z; maximum injection time (maxIT) of 250 ms; 100% normalized AGC target fragmented by HCD with 33% collision energy; 3 second cycle time; and exclusion of charge state <2 and >4.

Enriched tyrosine phosphopeptides were direct-loaded onto the analytical column as above, and were analyzed using an Exploris 480 Hybrid Quadrupole-Orbitrap mass spectrometer (Thermo Fisher Scientific) coupled to an Agilent 1260 LC system. Fractionated serine and threonine phosphopeptides were loaded onto in-house packed precolumn connected in tandem to an in-house packed analytical column, as described above. Peptides were separated using a 145 min gradient (11% for 10 min, 11-32% for 105 min, 32-60% for 10 min, 60-100% for 10 min, hold for 3 min, 100% to 0% for 7 min) with 70% acetonitrile in 0.2M acetic acid at flow rate of 0.2 mL/min with approximate pre-column split of 2000:1. Exploris was operated in data-dependent acquisition for MS1 scans with 350-2000 m/z scan range, 60,000 resolution, normalized AGC target of 300%, maxIT of 50 ms. For every full scan, MS2 spectra were collected with an isolation width of 0.4 m/z, maxIT of 250 ms, standard AGC target, fragmentation by HCD with 33% collision energy, resolution of 60,000, 3 second cycle time and dynamic exclusion (exclude for 45 sec if precursor occurs twice within 30 sec).

### Mass spectrometry data analysis

Mass spectra were analyzed using Proteome Discoverer (v2.5, Thermo Fisher Scientific) and searched using Mascot (v2.4) against the mouse Swiss-Prot database (v2021_03). For peptide-bound MHC, peptides were searched with no enzyme and variable methionine oxidation. Peptide spectrum matches were filtered by an ion score ≥15, length 8-11, search engine rank of 1, and aggregated across unique peptides. GibbsCluster 2.0 was used for motif analysis (26). For phosphoproteomic data, peptide spectrum matches were filtered by an ion score ≥20 for pTyr data and ≥25 for pSer/pThr data and search engine rank of 1. Missing values were converted to 1000 for downstream analysis. Data were processed in R studio (v4.1.0). Volcano plots were plotted with EnhancedVolcano and heatmaps were plotted with ComplexHeatmapR package. Global phosphoproteome data were subjected to PTM Signature Enrichment Analysis (27). KinMap was used for plotting kinases that were differentially phosphorylated (28). Protein expression between samples was visualized using a heatmap, in which Z-score normalization was applied to the matrix of protein MS expression values, bounded to 1 if normalized value > 1 and to -1 if normalized value < -1, and then scaled to a range of 0 to 1 (by adding 1 to all counts and dividing by 2).

### Immunohistochemical staining

GL261-hCD19-luc2 and CT2A-hCD19-luc tumor-bearing brains were harvested and fixed in 4% paraformaldehyde for 24 hours, paraffin-embedded, sectioned, and stained using standard hematoxylin & eosin stain and immunohistochemical methods, as previously described (29). Slides were scanned in brightfield on a Zeiss AxioScan.Z1 using a 20x objective. Antibodies are detailed in the **Supplemental Antibodies Table**.

#### Immunofluorescent staining

Formalin-fixed paraffin-embedded tissue sections of brains implanted with GL261-luc2 or CT2A-luc tumors were counterstained and immunolabeled using a 1:5000 dilution of Hoechst dye (10 mg/ml stock) in Odyssey Blocking Buffer to which anti-vimentin (AF594-conjugated, clone: D21H3; Cell Signaling #7675S) and anti-Ki67 (AF488-conjugated, clone: D3B5; Cell Signaling #11882S) primary antibodies were added at 1:25 dilutions. Tissue sections were incubated with the resulting counterstain/antibody solution for 1 hour in the dark at room temperature, rinsed in opaque Coplin jars containing fresh 1X PBS for 10 minutes in triplicate, and cover slipped in a 50% v/v glycerol solution diluted in 1X PBS immediately prior to imaging. Image tiles were acquired using a CyteFinder slide-scanning fluorescence microscope (RareCyte Inc.) at 20x magnification with 2x2 binning then stitched, registered, and flatfield-corrected using the MCMICRO image processing pipeline to generate whole-slide mosaic images. Tissue sections were also stained with hematoxylin and eosin for histological evaluation.

### Western blot

GL261-luc2 and CT2A-luc cells were seeded at 500,000 cells/well in 6-well plates and cultured for 48 hours with or without 50 ng/mL of recombinant murine IFNγ (#315-05, Peprotech) at 37°C in a humidified incubator with 5% CO2, and then lysed in RIPA buffer (#89900, Thermo Fisher Scientific) containing protease and phosphatase inhibitors (#11836153001, #524624; Sigma-Aldrich). SDS polyacrylamide gel electrophoresis was performed using 10 μg of each lysate boiled for 10 minutes in Laemmli sample buffer. Proteins were transferred onto a PVDF membrane using a wet Trans-Blot transfer system (Bio-Rad Laboratories), blocked with TBS buffer containing 3% milk for 1 hour at room temperature, incubated with primary antibody overnight at 4 °C, washed, and then incubated with rabbit secondary antibody (# NA934, GE Healthcare). Primary and secondary antibody dilutions were prepared with a TBS buffer containing 0.1% Tween20 and 3% milk. Staining was detected using Supersignal West Pico or Femto Chemiluminescent Substrate (Thermo Fisher Scientific) and Biorad ChemiDoc MP imaging system. Antibodies are detailed in the Supplemental Antibodies Table. Band densities were normalized to the sample’s corresponding B-actin band signal, averaged across experimental replicates, and compared between experimental conditions using AzureSpot Pro 1.4.

### Secreted protein and MHC expression analysis

GL261-luc2 and CT2A-luc were seeded at 1×10^6^ cells/well in 6-well plates with their respective media conditions, and treated with either none or 50 ng/mL of recombinant murine IFN-γ (BioLegend #575304) at 37°C in triplicate. The manufacturer-reported specific activity of the IFN-γ was 1-4×10^6^ units/mg. After 24 hours, the conditioned media were aspirated and frozen for storage. Wells were rinsed with PBS and the cells were detached by incubation with 0.5 mM EDTA in PBS. Cells were suspended in media, pelleted, and washed with media. Cells were then stained for 15 minutes with the Zombie Aqua Fixable Viability Kit (BioLegend) for live/dead discrimination. Samples were then washed, blocked with anti-mouse CD16/32 for 10 minutes and stained with fluorophore-conjugated antibodies (either H2-K^b^ with I-A/I-E, or H2-D^b^ alone) or respective isotype controls for 20 minutes on ice. PBS with 2% fetal bovine serum was used for antibody staining and washing. All antibodies were used at 1:100 dilution and detailed in the Supplemental Antibodies Table. Concurrently, splenocytes from a naïve mouse were dissociated, lysed with ACK buffer, and used as additional positive controls. Samples were washed and analyzed with a BD LSR Fortessa. Data were collected using FACSDiva (BD Biosciences) and then compensated and analyzed using FlowJo (v10, BD Biosciences).

For assessment of cytokine and chemokine signatures, the conditioned media were thawed, centrifuged at 3000 x *g* for 5 min to remove debris, and processed using the LEGENDplex bead-based immunoassay with cytokine and chemokine analyte panels (BioLegend #740446 Mouse Inflammation Panel and #740451 Mouse Proinflammatory Chemokine Panel) following the manufacturer’s instructions. Samples were acquired with FACSymphony A3 cell analyzer (BD Biosciences) and analyzed using the LEGENDplex Data Analysis Software Suite.

### Comparison of RNA sequencing from murine tumor and human tumor samples

Scaled TPM matrices across TCGA human cancer cohorts were obtained from the Broad GDAC Firehose (Firehose 2016_01_28 run) and multiplied by 1,000,000 to generate standard TPM values. The TPM matrices were previously created by aggregating RSEM gene-level outputs generated from bulk RNAseq data. Because the original read counts were unavailable, the TPM values were rounded to the nearest integers in order to simulate count data for use with DESeq2 (30). Using the same method, TPM matrices were generated from the GL261 and CT2A bulk RNAseq data. For comparability between human and mouse data, the orthologous genes shared by both species were identified from Ensembl Project’s “Multiple Species Comparison” function (Ensembl 102) and analyzed. Principal component analysis (PCA) and differential expression using DESeq2 were performed using default parameters. The top 500 differentially expressed genes (FDR-adjusted p < 0.05) were excluded from the PCA analysis to minimize species-specific differences and batch effects. As a metric for the overall transcriptomic similarity of the mouse models to each of the human cancers, the Euclidean distances in PCA-space were computed from the center of the mouse cohorts to the center of each human cohort. To visualize individual sample-to-sample differences and orthogonally confirm PCA clustering, clustered heatmaps were generated with the pheatmap package (v1.0.12) in R 4.1.1 (31). Representative histological images of human tumors were acquired from the Cancer Digital Slide Archive in cBioPortal (32, 33).

### Statistical analysis

Overall survival was measured from tumor implantation, estimated using Kaplan-Meier techniques and analyzed using logrank test with Bonferroni correction. Continuous variables were assessed using one-way ANOVA with the Holm-Šídák method to adjust for multiple comparisons. False discovery rates were calculated using Benjamini-Hochberg method. Analyses were performed with R, GraphPad Prism (v9.3.1), and Stata (v17.1).

## Results

### GL261-luc2 and CT2A-luc exhibit distinct biologic behavior, histology, and transcriptional profiles

**Figure 1:**
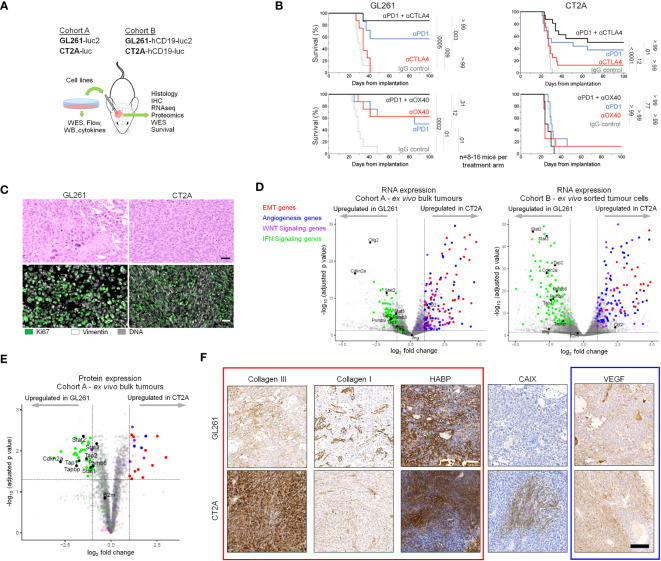
GL261-luc2 and CT2A-luc exhibit distinct biologic behaviors, histologies, and transcriptional profiles. **(A)** Schematic of the experimental analyses. Cohort A consisted of *in vitro* and bulk *ex vivo* samples of GL261-luc2 and CT2A-luc. Cohort B consisted of GL261-hCD19-luc2 and CT2A-hCD19-luc, in which human CD19 expression permitted the *ex vivo* sorting of hCD19-positive tumor cells. WES, whole exome sequencing; WB, Western Blot; Flow, flow cytometry; IHC, immunohistochemistry. **(B)** Kaplan-Meier overall survival curves associated with checkpoint immunotherapy in intracranial GL261-luc2 (*left*) and CT2A-luc (*right*) tumor-bearing mice. *Top:* anti-PD-1 and/or anti-CTLA-4 treatment experiments. *Bottom:* anti-PD-1 and/or anti-OX40 treatment experiments. (n=8-16 mice per experimental arm). One mouse in the single-agent anti-PD-1 GL261-luc2 group from the anti-CTLA-4 experiment was excluded due to tumor-unrelated death (day 9) prior to completing treatment. Adjusted p values are displayed from pairwise logrank tests, using Bonferroni correction for the 5 comparisons in each experiment. A two-sided adjusted p<0.05 for each experiment was considered significant. Checkpoint immunotherapy and IgG control dosing are detailed in the Methods. **(C)** Representative hematoxylin & eosin histological (*top*) and immunofluorescent (*bottom*) staining of *ex vivo* GL261-luc2 and CT2A-luc tumors. Scale bars = 50 µm. **(D)** *Left:* Volcano plot displaying the genes that were differentially expressed in *ex vivo* CT2A-luc bulk tumors, as compared to GL261-luc2 (n=4 mice each). *Right:* Volcano plot displaying the genes that were differentially expressed in *ex vivo* CT2A-hCD19-luc sorted tumor cells, as compared to GL261-hCD19-luc2 (n=3-5 mice each). Cutoffs included |log_2_FoldChange| >1 and Benjamini-Hochberg FDR-adjusted p<0.05. **(E)** Volcano plot displaying the proteins that were differentially expressed in *ex vivo* CT2A-luc bulk tumors, as compared to GL261-luc2 (n=3 mice each). Cutoffs included |log_2_FoldChange| >1 and Benjamini-Hochberg FDR-adjusted p<0.05. **(F)** Representative immunohistochemical staining of collagen III, collagen I, hyaluronan-binding protein (HABP), vascular endothelial growth factor (VEGF), and carbonic anhydrase IX (CAIX) in *ex vivo* GL261-hCD19-luc2 (*top*) and CT2A-hCD19-luc (*bottom*) tumors. Scale bar = 100 µm.

To identify the intrinsic mechanisms of immunotherapy response and resistance in the GL261 and CT2A murine models, respectively, we systematically compared their proteogenomic profiles. Unless otherwise specified, all experiments were conducted on *ex vivo* tumor samples. The tumor lines were transduced with firefly luciferase to permit the tracking of intracranial tumor growth *in vivo* (Cohort A, **Figure 1A**). We further extended our analysis of tumor cell-specific attributes through the evaluation of a second set of experiments, in which the immunologically inert human *CD19* was ectopically expressed in the GL261-luc2 and CT2A-luc lines to provide a tumor-specific marker that facilitated cell sorting and characterization of the tumor cells (Cohort B, **Figure 1A**).

As expected, we confirmed the sensitivity of GL261-luc to single-agent anti-PD-1 and single-agent anti-OX40, as well as enhanced benefit for anti-PD-1 plus anti-CTLA-4 or anti-OX40 combinatorial therapy in Cohort A mice (all p_adjusted_ ≤ 0.01 compared to IgG control; **Figure 1B**). In contrast, CT2A-luc demonstrated relative resistance with minimal benefit to single-agent immune checkpoint therapy (all p_adjusted_ ≥ 0.12 compared to IgG controls) and improved survival was only seen with anti-PD-1 plus anti-CTLA-4 therapy (p_adjusted_ < 0.001 compared to IgG control). The corresponding tumor growth plots are displayed in **Supplementary Figure 1**.

GL261-luc2 tumors were histopathologically characterized by polymorphic, poorly-differentiated cells with marked pleomorphism and sporadic giant cell features (**Figure 1C**, **Supplementary Figure 2A**). By contrast, CT2A-luc tumors displayed a spindled cellular morphology and fascicular architecture that were consistent with mesenchymal differentiation. Although both models had occasional foci of necrosis, neither displayed the diffuse infiltrative patterns or microvascular proliferation that are diagnostic for huGBM. Furthermore, glial fibrillary acidic protein (GFAP) – an intermediate filament expressed by astrocytic lineages, including a majority of huGBM – was only focally expressed in both tumor models (**Supplementary Figure 2B**).

We investigated the transcriptional differences between CT2A-luc and GL261-luc2 *ex vivo* tumors in Cohorts A and B and found that many genes were differentially expressed between the models (Cohort A: 24.9% of detected genes, Cohort B: 46.4% of detected genes; FDR-adjusted p<0.05; **Figure 1D**; **Supplementary Figure 3A**; **Supplementary Tables 1**, **2**). Among these genes, gene sets associated with epithelial-to-mesenchymal transformation (EMT), angiogenesis, and WNT signaling were notably enriched in CT2A tumors in both Cohorts, whereas interferon γ and α response pathways were enriched in GL261 tumors (FDR-adjusted p<0.1) – findings which were also reflected at the protein expression level (**Figure 1E**; **Supplementary Tables 3**, **4**). Because previous studies suggested that cell lines grown as neurospheres might better model huGBM (13, 23, 34), we also evaluated neurosphere-derived tumors and again found enrichment of EMT and WNT signaling gene sets in CT2A-hCD19-luc and inflammatory response-related gene sets enriched in GL261-hCD19-luc (**Supplementary Figure 3B**). EMT-related signaling pathways (e.g., TGF-β) were also enriched in CT2A-luc tumors. Because EMT is associated with marked remodeling of the extracellular matrix, we examined the extracellular matrix of *ex vivo* tumors using immunohistochemistry and qualitatively observed elevated deposition of collagen III and collagen I in the microenvironment of CT2A-luc tumors (**Figure 1F**; **Supplementary Figures 2C–E**). Additionally, gene sets associated with hypoxia were also enriched in the CT2A-luc tumors, which corresponded with a focally increased expression of carbonic anhydrase IX by immunohistochemistry (**Figure 1F**; **Supplementary Figures 2F, G**).

### Genomic profiles of GL261-luc2 and CT2A-luc

**Figure 2:**
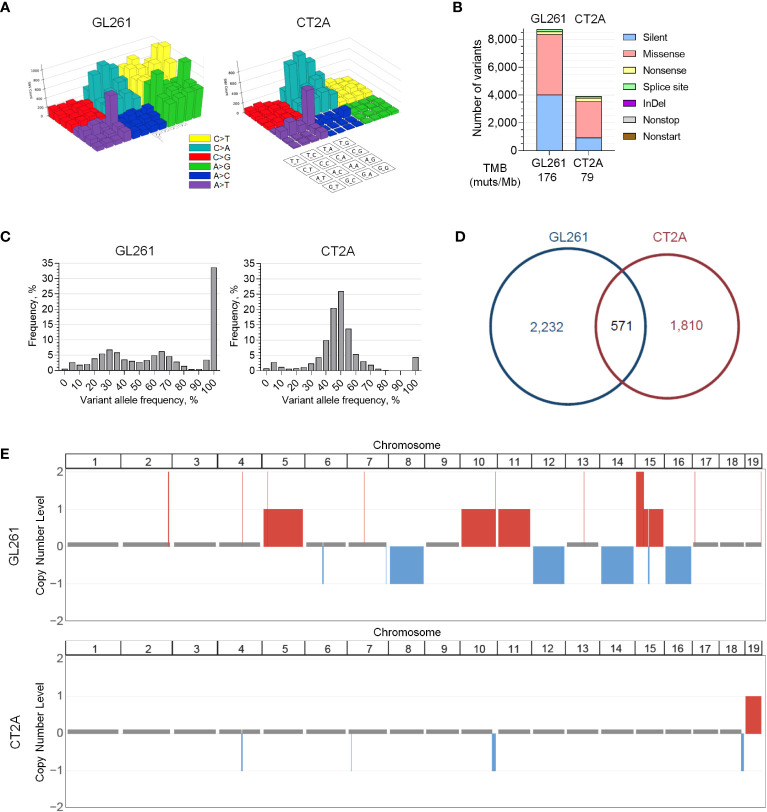
Genomic profiles of GL261-luc2 and CT2A-luc. **(A)** Lego plots visualizing the patterns of all types of transversion and transition mutations detected in whole exome sequencing of *in vitro* GL261-luc2 and CT2A-luc cells. Both models exhibited the C>A/G>T and CAG>CTG/GTC>GAC mutations that have been associated with a methylcholanthrene-induced etiology. GL261-luc2 additionally showed high levels of A>G/T>C and C>T/G>A transitions. **(B)** Frequency of small somatic sequence variants (*i.e.*, single nucleotide variants and small insertions/deletions [InDel]) by mutation type from whole exome sequencing of *in vitro* GL261-luc2 and CT2A-luc cells, with corresponding estimated tumor mutational burden (TMB). **(C)** Frequency of variants by variant allele fraction (VAF) from whole exome sequencing of *in vitro* GL261-luc2 and CT2A-luc cells. GL261-luc2 demonstrated an increased frequency of variants at 100% VAF (*i.e.*, likely homozygous). **(D)** Pie chart depicting the overlap of genes that have sequence variants (VAF ≥ 20%) between *in vitro* GL261-luc2 and CT2A-luc cells. **(E)** Copy number analysis of *in vitro* GL261-luc2 (n=5) and CT2A-luc (n=2) samples displaying somatic chromosomal segments that were significantly gained (*red*) or lost (*blue*) as compared to diploid reference (GISTIC2.0 FDR-adjusted p<0.05).

To investigate the potential genetic correlates to the histologic and transcriptional profiles that were observed in CT2A-luc, we performed whole-exome sequencing (WES) of both lines. Both models exhibited the canonical C>A/G>T transversion signature and CAG>CTG peak profile associated with a methylcholanthrene-induced etiology, which most closely resembles smoking carcinogen-related COSMIC Signature 4 in humans (35). GL261-luc2 also exhibited more C>T and A>G variants than CT2A-luc (**Figure 2A**) (36). Although CT2A-luc had less than half of the tumor mutational burden of GL261-luc2, both models were markedly hypermutated (approximately 79 and 176 mutations/MB, respectively), and both exhibited a predominance of missense single nucleotide variants and limited insertion/deletion burden (**Figure 2B**). Similar WES results were observed in non-luciferized GL261 and CT2A lines (data not shown). Excluding somatic variants with subclonal variant allele frequencies (VAF) <20%, among the 2,803 genes with variants in GL261-luc2 and 2,381 in CT2A-luc, 571 (12.4%) of altered genes were shared between the lines, but none of these involved known cancer drivers (**Figures 2C, D**; **Supplementary Table 5**). The copy number profiles of the models were clearly distinct. GL261-luc2 exhibited multiple whole chromosomal gains (e.g., chromosomes 5, 10, 11, 15) and losses (e.g., chromosomes 8, 12, 14,16) – among other segmental alterations – whereas CT2A-luc exhibited losses of chromosomal segments involving 4qC4, 7qA1, 10qD2-10qD3, and 18qE4 (all FDR-adjusted p<0.05; **Figure 2E**; **Supplementary Table 6**). In CT2A-luc, the 4qC4 loss included single-copy loss of *Cdkn2a/b*. GL261-luc2 distinctly harbored a *Kras* p.G12C clonal mutation whereas CT2A-luc had an *Nras* p.Q61L clonal mutation (**Supplementary Figure 3C**). *Nf1* and *Trp53* alterations were present in both models, but neither model exhibited *Idh1/2*, *Atrx*, *Braf*, *H3f3a* mutations nor copy number alterations of *Pten*, *Egfr*, *Nf1*, or *Rb1*, all of which are commonly associated with huGBM and astrocytoma.

### Multifactorial defects in antigen processing and presentation machinery in CT2A-luc

**Figure 3:**
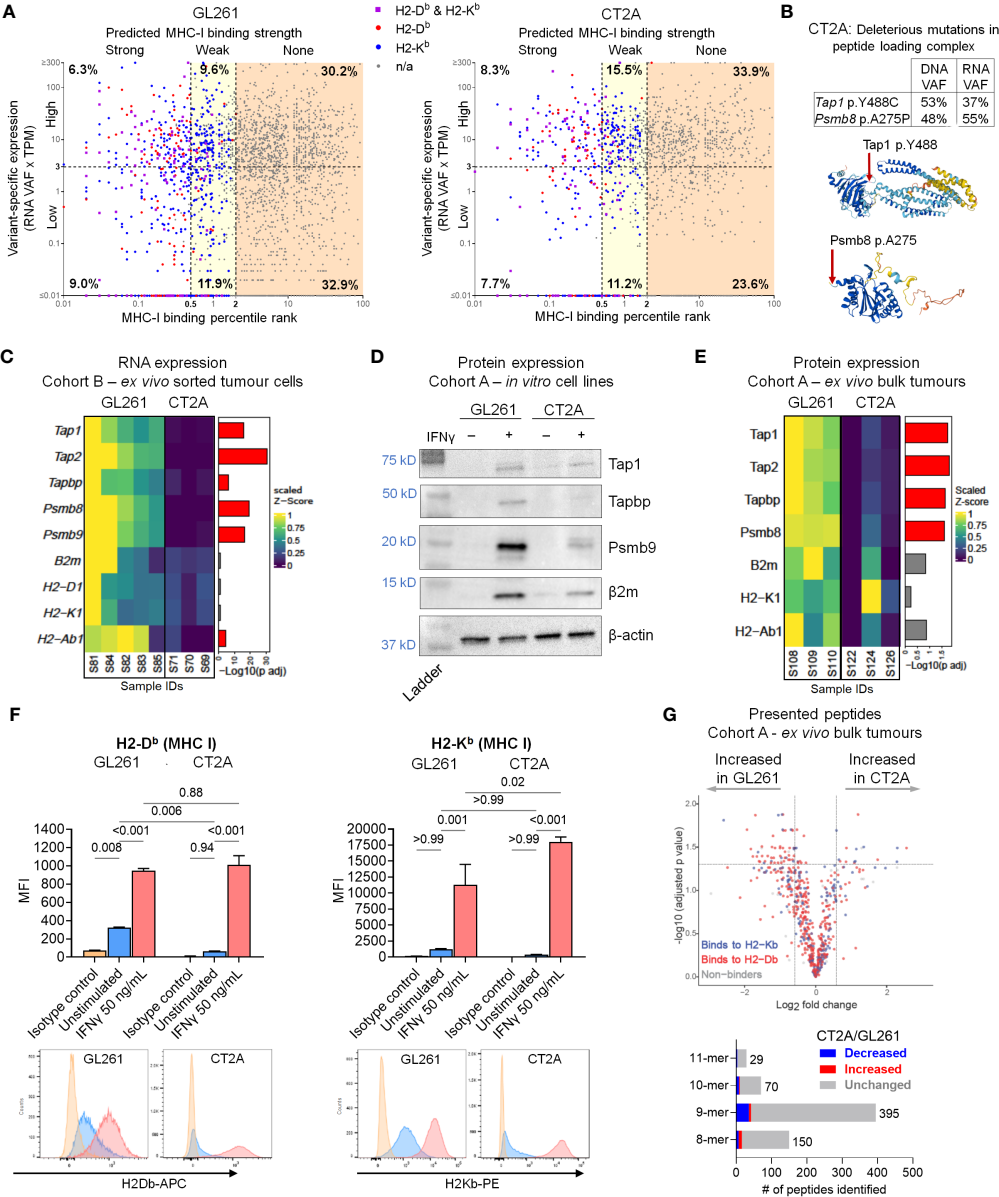
Multifactorial defects in antigen processing and presentation machinery in CT2A-luc. **(A)** Scatter plot displaying the predicted MHC class I binding strength (binding percentile rank) by variant-specific RNA expression (RNA variant allele frequencies [VAF] x TPM of gene’s expression) for each variant detected in the whole exome sequencing of GL261-luc2 (*left*) and CT2A-luc (*right*) tumors, colored by which MHC class I allele(s) the variant was predicted to bind. Axes are in log_10_ scale. Variant-specific expression was dichotomized into high and low using a cutoff of 3 TPM. MHC class I binding strength was categorized as strong (percentile rank < 0.5), weak (0.5 ≤ percentile rank < 2.0), or none (percentile rank ≥ 2.0). The corresponding percent of total variants found in each cell is displayed. TPM = transcripts per million. **(B)** *Top*: The VAF of antigen presentation machinery gene mutations detected in the whole exome sequencing of *in vitro* CT2A-luc and RNA sequencing of CT2A-luc tumors. *Bottom*: The predicted 3-D structure of Tap1 (Y488 residue highlighted) and Psmb8 (A275 residue highlighted) from AlphaFold. **(C)** Heatmap depicting the differential RNA expression of antigen processing and presentation machinery genes in *ex vivo* sorted GL261-hCD19-luc2 (n=5 mice) and CT2A-hCD19-luc (n=3 mice) tumor cells, with the corresponding FDR-adjusted p value. Expression values were row normalized, Z-scored, bounded, and scaled. Red = FDR-adjusted p value<0.05. **(D)** Western blot displaying the antigen presentation and processing machinery protein expression in *in vitro* GL261-luc2 and CT2A-luc cell lines, with or without 50 ng/mL IFN-γ stimulation. β-actin was evaluated as a loading control. Displaying one representative of two replicate experiments (replicates shown in **Supplementary File**). Corresponding band densitometry quantification is shown in **Supplementary Figure 4B**. **(E)** Heatmap depicting the differential protein expression of antigen processing and presentation machinery genes in *ex vivo* bulk GL261-luc2 and CT2A-luc tumors (n=3 mice each), with the corresponding FDR-adjusted p value. Expression values were row normalized, Z-scored, bounded, and scaled. Red = FDR-adjusted p value<0.05. **(F)** *Top*: MHC class I surface expression median fluorescence intensity (MFI) detected by flow cytometric analysis on *in vitro* GL261-luc2 and CT2A-luc cells that were either stimulated with 50 ng/mL IFN-γ or unstimulated for 24 hours, compared to isotype controls. Expression was analyzed using one-way ANOVA, with two-sided pairwise p values adjusted for multiple testing using the Holm-Šídák method. The experiment was conducted in triplicate, bars = mean ± standard error. *Bottom*: Representative histograms of MHC expression. **(G)** *Top:* Volcano plot displaying the differential presentation of peptides between *ex vivo* GL261-luc2 and CT2A-luc bulk tumors (n=3 mice each), colored by MHC class I allele. *Bottom:* the proportions of presented peptides that were significantly decreased (blue) or increased (red) in *ex vivo* CT2A-luc bulk tumors as compared to GL261-luc2.

The availability of high-quality MHC class I neoantigen candidates did not appear to differ between the two lines, as they both demonstrated similar proportions of highly-expressed strong predicted HLA class I binders (**Figure 3A**). On the other hand, CT2A-luc uniquely contained multiple mutations in antigen presentation machinery genes that were computationally predicted to have deleterious biologic effects, including a clonal p.A275P missense mutation in *Psmb8* (a subunit of the immunoproteasome, which degrades proteins into peptides for loading onto MHC class I) and a clonal p.Y488C missense mutation in *Tap1* (which transports peptides into the endoplasmic reticulum for loading onto MHC class I) (**Figure 3B**). Based on these results and the critical role that MHC molecules play in mediating immune responses, we next experimentally examined the expression of antigen processing and presentation machinery components in CT2A-luc.

Although RNA sequencing of *ex vivo* bulk tumor and sorted tumor cells showed similar expression of the MHC class I α (*H2-D1* and *H2-K1*) and β (*B2m*) chains between CT2A-luc and GL261-luc2, CT2A-luc displayed lower expression of genes associated with the immunoproteasome complex (*Psmb8*, *Psmb9*) and peptide transporter/loading complex (*Tap1*, *Tap2*, *Tapbp*) (differential expression analysis, all FDR-adjusted p<0.05; **Figure 3C**; **Supplementary Figure 4A**). Both GL261-luc2 and CT2A-luc lines exhibited minimal basal levels of antigen presentation and processing machinery protein expression by Western blot analyses of *in vitro* cultured cells (**Figure 3D**; **Supplementary Figure 4B**). Proteomic analysis of *ex vivo* bulk tumors further confirmed that Tap1, Tap2, Tapbp, and Psmb8 protein expression was reduced in CT2A-luc tumors (all FDR-adjusted p<0.05, **Figure 3E**; **Supplementary Table 7**).

Deficiency in the peptide loading complex may limit the peptides available for binding to MHC class I, and accordingly, the successful assembly of MHC class I molecules for surface expression (37). Indeed, flow cytometric analysis confirmed considerably less MHC class I (H2-D^b^) surface expression on CT2A-luc *in vitro* (p_adjusted_=0.006; **Figure 3F**) compared to GL261-luc2. Without IFN-γ stimulation, GL261-luc2 cells displayed surface expression of H2-D^b^ (p_adjusted_ = 0.008 compared to isotype control), but not H2-K^b^ (p_adjusted_ > 0.99 compared to isotype control); whereas neither H2-D^b^ nor H2-K^b^ cell surface expression were detected on CT2A-luc cells at baseline (both p_adjusted_ ≥ 0.94 compared to isotype control; **Figure 3F**). Furthermore, of 644 MHC class I-bound peptides immunoprecipitated from *ex vivo* tumors, 64 (9.9%) peptides were more likely to be presented by GL261-luc2, whereas 16 (2.5%) peptides were more likely to be presented by CT2A-luc – although this analysis was complicated by the presence of infiltrating non-neoplastic cells with MHC expression (**Figure 3G**; **Supplementary Figure 4C**, **Supplementary Table 8**). Beyond MHC class I, both cell lines had low expression of β2m and minimal expression of MHC class II *in vitro* (all p_adjusted_>0.05 compared to isotype control; **Supplementary Figures 4D, E**), with CT2A-luc exhibiting minimal MHC class II expression (p_adjusted_ = 0.04). Taken together, these data suggest a marked, multi-factorial defect in antigen presentation by CT2A-luc tumors; whereas GL261-luc2 tumors exhibited intact antigen presentation machinery.

### CT2A-luc is deficient in interferon response and signaling

**Figure 4:**
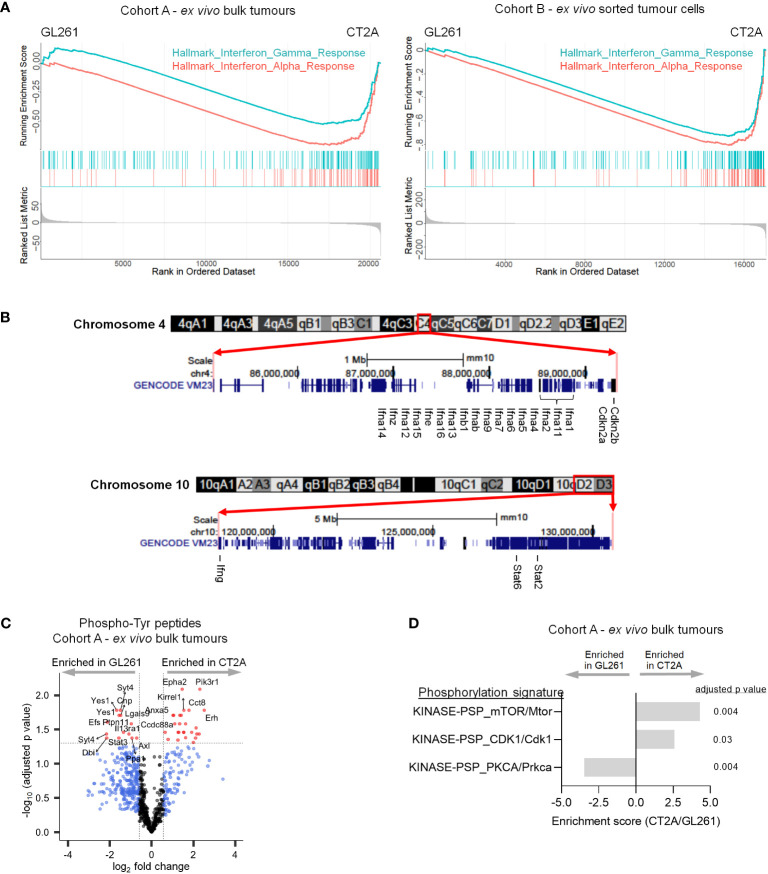
CT2A-luc is deficient in interferon response and signaling. **(A)** Gene set enrichment plots derived from the differential expression analyses in **Figure 1E**, displaying hallmark interferon response gene sets that were significantly depleted in CT2A-luc as compared to GL261-luc2, for *ex vivo* bulk tumors (*left*) and sorted hCD19+ tumor cells (*right*). n=3-5 mice each. **(B)** Chromosomal ideograms with GENCODE VM23 tracks for the chromosomal segments involving 4qC4 (*top*) and 10qD2-10qD3 (*bottom*) that were lost in CT2A-luc tumors, from the UCSC Genome Browser (http://genome.ucsc.edu). Select genes are highlighted. **(C)** Volcano plot displaying differential phosphorylation of tyrosine residues between *ex vivo* GL261-luc2 and CT2A-luc bulk tumors (n=856 total phosphotyrosine [pTyr] peptides). Cutoffs included |log_2_FoldChange| > log_2_(1.5) and Benjamini-Hochberg FDR-adjusted p<0.05. n=3 mice each. **(D)** Post-translational modification Signature Enrichment Analysis (PTM-SEA) of the differentially expressed phosphoserine and phosphothreonine peptides between *ex vivo* GL261-luc2 and CT2A-luc bulk tumors. FDR-adjusted p<0.05. n=3 mice each.

Although multiple pathways were enriched in CT2A-luc tumors, only two gene sets were consistently downregulated in CT2A-luc in both Cohorts: interferon (IFN)-α and IFN-γ response via both RNA and protein expression analyses (**Figure 4A**). To determine if there was a genomic basis for this altered circuitry, we evaluated the multiple arm-level chromosomal copy number alterations that were detected in each cell line through analysis of WES data. CT2A-luc uniquely exhibited a single-copy loss of a chromosomal segment involving 4qC4 (FDR-adjusted p=0.04), which encompassed multiple type I IFN genes, as well as a single-copy loss of 10qD2-10qD3 (FDR-adjusted p=0.04), which contained *Stat2*, *Stat6*, and *Ifng* (**Figures 2E**; **4B**, **Supplementary Table 6**).

Consistent with our observation of down-regulated IFN response pathways in CT2A-luc, phosphoproteomic analysis revealed decreased phosphorylation of several members of the JAK/STAT pathway in *ex vivo* CT2A-luc tumors, including Ptpn11 (i.e., Shp2), Il13ra1, and Stat3 – together suggesting reduced JAK/STAT signaling (**Figure 4C**; **Supplementary Figure 5A**, **Supplementary Table 9**). CT2A-luc tumors were further distinguished by phosphorylation of the Pik3 regulatory subunit 1 (Pik3r1) and enrichment of downstream mTOR signaling, consistent with a parallel activation of the Pi3k/Akt/mTOR pathway (**Figures 4C, D**; **Supplementary Table 10**). CT2A-luc also displayed elevated phosphorylation of cell cycle (Cdk1) and decreased phosphorylation of Prkca pathways, which are involved in diverse cellular signaling pathways (**Figure 4D**; **Supplementary Figures 5B, C**).

During immune responses, IFN-γ strongly upregulates the antigen processing and presentation components in cells (38) – which we hypothesized might be impaired in CT2A-luc. IFN-γ treatment boosted the expression of antigen processing and presentation proteins (e.g., Tap1, Tapbp, Psmb9, β2m) in both cell lines *in vitro*, although notably to a lesser degree in CT2A-luc (**Figure 3D**; **Supplementary Figure 4B**). However, MHC class I surface expression was strongly upregulated by IFN-γ treatment in both lines (all p_adjusted_ ≤ 0.001; **Figure 3F**; **Supplementary Figure 4D**), potentially suggesting that the Tap1 mutational defect and impaired antigen presentation machinery in CT2A could be – at least partially – overcome by exposure to exogenous IFN-γ. Analysis of the RNA sequencing data revealed that *ex vivo* purified CT2A-hCD19-luc tumors retained IFN-γ receptor expression (*Ifngr1* log_2_FoldChange 0.39, FDR-adjusted p=0.02; *Ifngr2* log_2_FoldChange 1.41, FDR-adjusted p = 5.38E-10) compared to GL261-hCD19-luc2 tumors (**Supplementary Table 2**).

### Secreted immunomodulatory proteins distinguish GL261-luc2 and CT2A-luc

**Figure 5:**
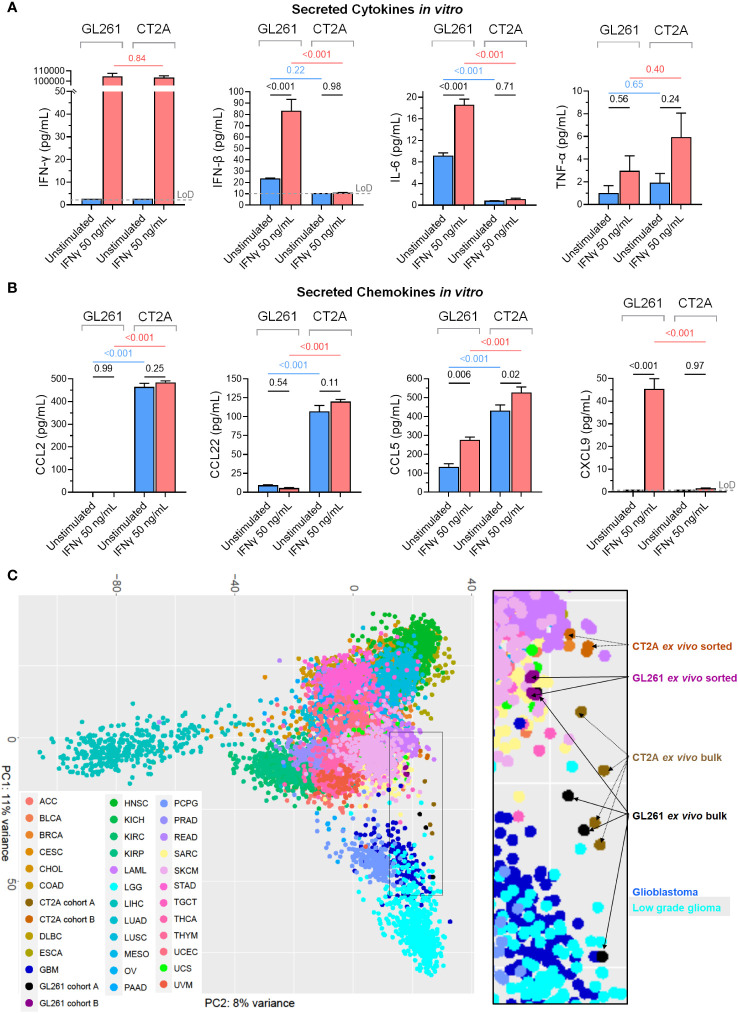
The secreted immunomodulatory protein profiles and relationship to human cancers of GL261-luc2 and CT2A-luc models. **(A, B)** Secreted **(A)** cytokines (IFN-γ, IFN-β, IL-6, TNF-α) and **(B)** chemokines (CCL2, CCL22, CCL5, CXCL9) were profiled from the conditioned media of the *in vitro* GL261-luc2 and CT2A-luc cultures from the **Figure 3F** experiment, which had been cultured for 24 hours without (*blue*) or with (*red*) IFN-γ (50 ng/mL). The experiment was conducted in triplicate, with secreted peptide concentrations graphed as mean ± standard error and compared using one-way ANOVA. The assay’s limit of detection (LoD; *grey dashed line*) was displayed and analyzed for samples whose values were above the LoD. For assessment of IFN-γ secretion, the IFNγ-stimulated samples still contained the experimentally administered IFN-γ. P values were adjusted for multiple testing using the Holm-Šídák method. The cell lines were also evaluated for IL-23, IL-10, GM-CSF, IL-17A, IL-1α, IL-1β, IL-12p70, IL-27, CCL3, CCL4, CXCL10, CCL20, CXCL1, CCL11, CCL17, CXCL5, and CXCL13; displayed in **Supplementary Figure 6**. **(C)** Unsupervised principal component analysis of whole transcriptome expression of the *ex vivo* bulk (Cohort A) and *ex vivo* tumor sorted (Cohort B) GL261-luc2 and CT2A-luc samples alongside RNA sequencing of all human cancer samples from TCGA. The 500 genes that were most differentially expressed between mouse and human tumor samples were excluded to help minimize species-level effects. Inset = higher magnification. OncoTree cancer type definitions were detailed previously (39). **Supplementary Figure 7A** shows the corresponding unsupervised principal component analysis without the exclusion of the 500 genes that were most differentially expressed between mouse and human tumor samples.

To assess how GL261-luc2 and CT2A-luc interact with the immune microenvironment, we profiled their secretion of 12 cytokines and 13 chemokines that are known to have important immunomodulatory roles. Unstimulated GL261-luc2 secreted the pro-inflammatory IL-6 and IFN-β cytokines, which were further increased following IFN-γ stimulation (**Figure 5A**). By contrast, unstimulated CT2A-luc only minimally secreted IL-6 and IFN-β; and these were unchanged upon IFN-γ stimulation, again suggesting impaired response to IFN-γ in CT2A-luc. Both lines lacked detectable IFN-γ, IL-17A, and GM-CSF secretion, and showed limited secretion of IL-10, IL-1β, and TNF-α (**Supplementary Figures 6A, B**). CT2A-luc demonstrated substantial baseline secretion of the CCL-2, CCL-5, and CCL-22 chemokines, all of which are known to play important roles as myeloid chemoattractants (**Figure 5B**), in marked contrast to GL261-luc2. Analysis of the *ex vivo* RNA sequencing data from Cohort A tumors also found increased *Ccl22* chemokine expression among CT2A-luc tumors (**Supplementary Figure 6C**).

Additionally, whereas the chemokines CCL4 (a natural killer cell and monocyte chemoattractant), CXCL10 (a broad immune cell population chemoattractant), CXCL9 (activated T cell chemoattractant), and CXCL1 (neutrophil chemoattractant) were secreted at low-to-negligible baseline levels in both models, their secretion was increased following IFN-γ treatment in GL261-luc2 (all p_adjusted_ < 0.05; **Figure 5B**; **Supplementary Figure 6B**). Neither line had detectable secretion of CCL3, CCL11, CCL17, CXCL5, or CXCL13 chemokines, including after IFN-γ stimulation (**Supplementary Figure 6B**). Chemokine gradients strongly influence the immune cell composition of the TME and prior studies have identified a myeloid cell predominance in CT2A tumors (17, 40). The murine Microenvironment Cell Population (mMCP) tool (24) was used to estimate the immune cell abundances from the RNA sequencing data from Cohort A *ex vivo* bulk tumors, and found a greater proportion of monocytes in CT2A-luc tumors as compared to GL261-luc2 tumors (log_2_FoldChange 1.03, p=0.03; **Supplementary Table 11**). In addition to secreted immunomodulatory proteins, RNA sequencing analysis of the *ex vivo* purified tumors showed that CT2A-hCD19-luc expressed less *Cd274* (i.e. PD-L1; log_2_FoldChange -2.27, FDR-adjusted p = 5.44E-13), but not *Pdcd1lg2* (i.e. PD-L2; log_2_FoldChange -0.48, FDR-adjusted p = 0.46) than GL261-hCD19-luc2 (**Supplementary Table 2**).

### The relationship of GL261-luc2 and CT2A-luc models to human cancer contexts

To investigate to what extent these murine tumor lines might transcriptionally model huGBM, we performed unsupervised principal component analysis that included huGBM (166 samples) as well as all other cancer types available in TCGA. We thereby attempted to assess the expression of all genes shared by both human and mouse transcriptomes (n=15,457 genes) (**Figure 5C**). The top 500 differentially expressed genes between mouse tumors and human tumors were excluded from the analysis to help account for species-specific transcriptional bias (as well as without exclusion in **Supplementary Figure 7A**). Both GL261-luc2 and CT2A-luc *ex vivo* samples occupied the transcriptional space between human gliomas (including glioblastoma and low-grade glioma) and other human cancer types (including cutaneous melanomas and sarcomas) in the first principal component of principal component analysis.

We assessed whether CT2A-luc may model a distinct human cancer context as compared to GL261-luc2 by evaluating the murine tumors against human cancers that commonly exhibit similar features to those that we observed in our GL261-luc2 and CT2A-luc characterizations, including RAS driver mutations (e.g., pancreatic adenocarcinoma and colorectal adenocarcinoma), carcinogen-induced mutation signatures (lung adenocarcinoma), and mesenchymal differentiation (renal cell carcinoma) – in addition to huGBM. Because CT2A-luc was characterized by notable dysregulation of epithelial-mesenchymal transition, angiogenesis, WNT signaling, and IFN-α/γ response hallmark gene sets, we repeated the unsupervised hierarchical clustering analyses using only the member genes of those hallmark gene sets (**Supplementary Tables 12**, **13**; **Supplementary Figures 7B–D**). From this analysis, all CT2A-luc samples clustered together and were more similar to seven (of 166) huGBM and two (of 534) kidney renal cell carcinoma samples, rather than to GL261-luc2. Review of the pathology reports and histological images from TCGA database for these huGBMs revealed that all indeed displayed mesenchymal differentiation (e.g., gliosarcomatous or spindle cell morphology) (**Supplementary Figure 7B**). Additionally, six of these seven huGBMs that had been previously analyzed by TCGA consortium have been classified into the mesenchymal subtype of huGBM. Comparison of the transcriptional profiles of these seven huGBMs to those of the 159 unrelated samples notably revealed downregulation of *TAP1* (LFC=-0.84, FDR-adjusted p=0.008; **Supplementary Table 14**). Similar to the seven huGBM samples, the pathology reports for both neighboring kidney cancer samples revealed a diagnosis of clear cell renal cell carcinoma with sarcomatoid features (i.e., mesenchymal differentiation).

## Discussion

Our genetic and histologic characterization of GL261-luc2 and CT2A-luc tumors revealed limited shared essential features with huGBM. Neither GL261-luc2 nor CT2A-luc models exhibited the diffusely infiltrative growth that is a defining hallmark of human diffuse gliomas including IDH-wildtype glioblastoma. Additionally, microvascular proliferation, *Tert* promoter mutations, *Egfr* amplification, or *Pten* loss (analogous to monosomy 10 in humans) – which are included as essential diagnostic criteria for WHO CNS grade 4 IDH-wildtype glioblastoma – were not observed in either model. Likewise, from the genetic perspective, while both models contained clonal hotspot mutations in RAS genes (*Kras* p.G12C in GL261-luc2 and *Nras* p.Q61L in CT2A-luc), which are important oncogenic drivers across multiple human cancers, such mutations have only been identified in <1% of huGBM tumors in TCGA. CT2A-luc did exhibit single-copy loss of *Cdkn2a/b*, although up to 40-50% of huGBMs have homozygous loss (41). These murine models demonstrated marked hypermutation, whereas most newly diagnosed and recurrent huGBMs demonstrate a modest tumor mutational burden (<10 mutations/MB) (42). Although huGBM patients with *de novo* hypermutation (i.e., as a result of germline DNA mismatch repair or POL-E deficiencies) arise occasionally and have been observed to respond to ICB, the more frequent condition of temozolomide-induced acquired hypermutation (noted in approximately 20% of recurrent huGBMs) has not been associated with a favorable response (42, 43).

To overcome the limitations of carcinogen-induced huGBM models, a diverse array of genetically engineered immunocompetent mouse models have been developed in recent years which more accurately recapitulate the molecular, histopathologic, and therapeutic features of huGBM – which have been reviewed elsewhere (13). However, the ability of such models to fully reflect the complex immunosuppressive TME and behavior of huGBM remains unclear. In this context, we found that the transcriptional profiles of GL261-luc2 and CT2A-luc tumors appear to more closely resembled human gliomas than other cancer types in TCGA – although the comparison of interspecies RNA sequencing data is beset by multiple limitations. Across our transcriptional and proteomic analyses, CT2A-luc was distinguished from GL261-luc2 by its mesenchymal differentiation and by its marked deficits in interferon response and antigen presentation pathways. In humans, several cancer types can manifest epithelial-mesenchymal transformation *de novo* or in response to treatment, including a subset of human IDH-wildtype glioblastomas that exhibit a mesenchymal histological subtype (i.e., gliosarcoma) and/or transcriptional profile and have been associated with worse survival (44, 45). In a large longitudinal analysis of gliomas (including 168 patients with RNA sequencing data for at least 2 timepoints), 38% and 45% of IDH-wild type diffuse gliomas displayed a mesenchymal cell state at initial and recurrent timepoints, respectively (45). We observed that multiple mesenchymal huGBMs from TCGA clustered more closely to CT2A-luc tumors than to other huGBMs, including a recurrent tumor that had acquired a mesenchymal cell state whereas its corresponding non-mesenchymal primary tumor clustered separately from CT2A-luc.

Mesenchymal huGBMs are also characterized by dense myeloid cell infiltrates, which have been well-described in the tumor microenvironment of CT2A (17, 40, 45, 46). For instance, using flow cytometric analysis, Liu et al. showed that tumor-associated macrophages comprise a substantially greater proportion (approximately 5-6x) of tumor-infiltrating CD45+ immune cells in CT2A tumors than GL261 tumors. Additionally, mass cytometry by time of flight (CyTOF) analyses by Khalsa et al. suggested that the CT2A TME features a greater proportion of resident macrophages (CD11b+ F4/80+ CD64+ Ly6C−) and infiltrating macrophages (CD11b+ F4/80+ CD64+ Ly6C+) than GL261. Single-cell RNA sequencing of TILs by Khan et al. found that GL261 is enriched with progenitor exhausted CD8+ T cells, whereas CT2A was enriched with terminally exhausted CD8+ T cells and regulatory CD4+ T cells (47). Although the tumors’ immune cell composition was not an aim of our study, analysis of our bulk tumors’ RNA profiles also identified a higher estimated proportion of monocytes in CT2A-luc than GL261-luc2. Consistent with these findings, we observed that CT2A-luc secreted multiple chemokines involved in myeloid cell and regulatory T cell chemoattraction in the huGBM tumor microenvironment (e.g., CCL-2 and CCL-22) (48, 49). In the tumor microenvironment of human gliomas, CCL2 has been shown to recruit both CCR4+ Treg and CCR2+ Ly6C+ monocytic myeloid-derived suppressive cells (48). Additionally, CCL22 has been shown to recruit differentiated Tregs into the glioblastoma TME (49).

The role of mesenchymal differentiation and response to immunotherapy is unclear in huGBM, with prior analyses of bulk RNA sequencing data identifying an association between the mesenchymal RNA subtype of huGBM and expression of both immune suppressive and proinflammatory gene signatures (50). However, when we compared the transcriptional profiles of CT2A-luc to human cancer samples, we identified a subset of mesenchymal huGBMs that indeed displayed a similar loss of antigen presentation and processing machinery – suggesting that mesenchymal glioblastomas may comprise a more complex spectrum of cell states with regards to immunotherapeutic resistance.

Whereas GL261 is sensitive to various immunotherapeutic modalities, CT2A is broadly resistant to single-agent immunotherapies aimed at T cell responses – including immune checkpoint inhibitors, vaccine therapy, and oncolytic virotherapy – which can be explained by our findings of deficits in antigen presentation machinery and interferon response in CT2A-luc (12, 17–21). The immunotherapeutic resistance of CT2A persists even with the ectopic expression of luciferase, which has been shown to confer increased immunogenicity to cell lines (51). Accordingly, ectopic expression of luciferase is a notable limitation of these models. Luciferase expression with bioluminescent imaging was used herein to ensure consistent tumor engraftment and sizes for all therapeutic and *ex vivo* experiments, and thereby avoid bias in our analyses due to differences in tumor engraftment or growth. An analysis of the tumor-immune microenvironment of GL261 tumors versus GL261-luc2 tumors found no significant differences in the presence of infiltrating immune cell populations (52).

Defects in antigen presentation machinery have been well-described across a spectrum of human cancer types – including mutations in *TAP1* and *PSMB8* like the ones we observed in CT2A-luc (53). Furthermore, in multiple cancer types such as melanoma (54–57), NSCLC (58), and Merkel cell carcinoma (59), the loss of MHC class I expression and defects in antigen presentation machinery or IFNy-response pathways have been recurrently associated with resistance (both intrinsic and acquired) to immunotherapy. Interestingly, we observed that CT2A-luc also clustered alongside several cutaneous melanoma tumors in transcriptional space. Melanoma is commonly characterized by carcinogen (i.e., ultraviolet light)-induced hypermutation and sensitivity to immune checkpoint inhibitors. However, only 20-50% of patients with advanced melanoma experience durable responses to immune checkpoint inhibitors (60, 61). Multiple mechanisms of immunotherapy resistance in melanoma have been elucidated (62), among them being MHC class I downregulation in conjunction with de-differentiation (including mesenchymal differentiation and angiogenesis upregulation) that have been associated with innate and acquired resistance to PD-1 checkpoint blockade (57, 63).

We found that CT2A-luc intrinsically shared these mechanisms, but we also observed that exogenous IFN-γ treatment could at least partially restore MHC class I expression in CT2A-luc – suggesting that select multi-pronged immunotherapeutic strategies may overcome CT2A’s inherent resistance to immunotherapy. Indeed, when PD-1 and CTLA-4 inhibitors were combined, we noted a modest therapeutic benefit in CT2A-luc tumor-bearing mice. In light of these findings, investigation of therapeutic combinations that address CT2A’s distinctive processes, such as immune contexture (e.g. myeloid-targeting immunotherapies), angiogenesis (e.g. bevacizumab), and mesenchymal phenotype (e.g. ritanserin) may help identify strategies that translate to the treatment of analogous cancer types in humans. Other studies have reported success with such combination approaches in CT2A, including PD-(L)1 inhibition with either adjuvanted neoantigen vaccination, bacterial antigen-armed oncolytic measles virotherapy, GITR agonist, or ectopic VEGF-C expression (17, 21, 64, 65). Building on our results, future studies that functionally dissect the individual contribution of each of the features detailed herein to CT2A’s overall resistance to immunotherapy will be informative. In particular, the enhancement of IFN-γ signaling warrants additional study for multi-modal therapeutic strategies in both CT2A and the human cancer contexts that it models. To overcome the obstacles posed by the blood-brain barrier, half-life in the interstitial fluid, and targeted localization to the tumor environment, such studies likely need to incorporate novel drug delivery technologies (e.g. convection-enhanced delivery, encapsulation in microspheres/nanoparticles, IFN-γ protein vs. mRNA delivery, etc.) or stimulation of IFN-γ release from existing cells in the tumor microenvironment. Pre-implantation stimulation of CT2A cells with IFN-γ also faces experimental challenges that should be taken into consideration, including if IFN-γ exposure leads to MHC class I upregulation *in vitro*, those CT2A tumors may be less likely to engraft in health mice and the effects of IFN-γ may only be transient.

Taken together, our findings indicate that although the clinical contexts that can be modeled by GL261 and CT2A for huGBM are limited, CT2A-luc may provide an informative preclinical model in immuno-oncology for investigating therapeutic strategies that can overcome immunotherapy resistance of cancers due to antigen presentation machinery loss, upregulated angiogenesis, and mesenchymal differentiation.

## Data availability statement

WES data were deposited to the SRA repository (PRJNA1056465) and RNA sequencing data were deposited to the GEO repository (GSE215123). Mass spectrometry data were deposited to the ProteomeXchange Consortium via the PRIDE partner repository as PXD036720.

## Ethics statement

Ethical approval was not required for the study involving humans in accordance with the local legislation and institutional requirements. Written informed consent to participate in this study was not required from the participants or the participants’ legal guardians/next of kin in accordance with the national legislation and the institutional requirements. The animal study was approved by Dana-Farber Cancer Institute and Harvard Medical School Animal Care and Use Committees. The study was conducted in accordance with the local legislation and institutional requirements.

## Author contributions

JI: Writing – original draft, Writing – review & editing, Conceptualization, Data curation, Formal analysis, Investigation, Methodology. NR: Conceptualization, Data curation, Formal analysis, Investigation, Methodology, Software, Validation, Visualization, Writing – review & editing. RA: Conceptualization, Formal analysis, Investigation, Writing – review & editing, Data curation. EP: Conceptualization, Investigation, Writing – review & editing, Data curation, Formal analysis, Methodology. PG: Conceptualization, Formal analysis, Investigation, Supervision, Visualization, Writing – review & editing, Data curation. MN: Conceptualization, Data curation, Formal analysis, Investigation, Visualization, Writing – original draft, Writing – review & editing, Validation. MS: Formal analysis, Investigation, Visualization, Writing – review & editing, Conceptualization, Data curation, Methodology. BE: Investigation, Writing – review & editing, Data curation, Validation. KS: Investigation, Writing – review & editing, Data curation. RP: Investigation, Writing – review & editing, Data curation, Formal analysis, Methodology. MD: Investigation, Writing – review & editing, Formal analysis, Methodology. SK: Investigation, Writing – review & editing, Formal analysis. KY: Investigation, Writing – review & editing, Formal analysis, Methodology. GB: Investigation, Writing – review & editing, Formal analysis, Methodology. RJ: Investigation, Writing – review & editing, Supervision. MS: Investigation, Writing – review & editing, Methodology, Supervision. DN: Supervision, Writing – review & editing, Methodology. FW: Methodology, Supervision, Investigation, Writing – review & editing, Conceptualization. EC: Investigation, Writing – review & editing, Conceptualization, Data curation, Funding acquisition, Methodology, Resources. GF: Investigation, Writing – review & editing, Conceptualization, Funding acquisition, Methodology, Resources, Supervision. AS: Investigation, Writing – review & editing, Conceptualization, Funding acquisition, Methodology, Resources, Supervision. CW: Conceptualization, Funding acquisition, Investigation, Methodology, Project administration, Supervision, Visualization, Writing – original draft, Writing – review & editing, Resources. DR: Conceptualization, Data curation, Funding acquisition, Investigation, Methodology, Project administration, Resources, Supervision, Visualization, Writing – original draft, Writing – review & editing.
