# Supplementary material for: Antigen presentation deficiency, mesenchymal differentiation, and resistance to immunotherapy in the murine syngeneic CT2A tumor model
Source: Front Immunol. 2023 Dec 28;14:1297932. doi: 10.3389/fimmu.2023.1297932 (PMC10782385; doi:10.3389/fimmu.2023.1297932)

SUPPLEMENTAL FIGURES AND LEGENDS

Supplemental Figure 1. Tumor growth of GL261-luc2 and CT2A-luc tumors

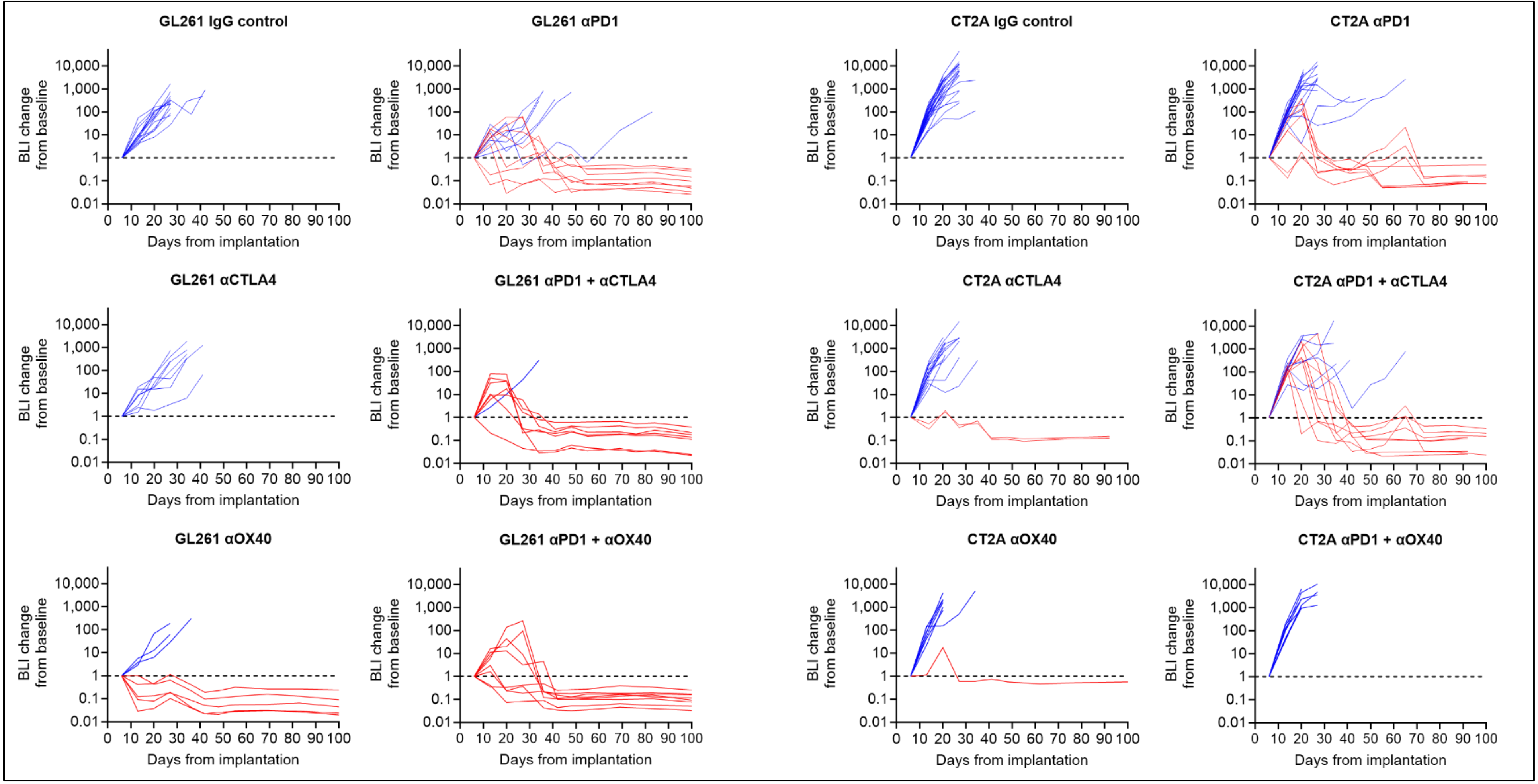

Spider plots of tumor growth in GL261-luc2 (left) and CT2A-luc (right)-bearing mice, as measured by bioluminescent imaging (BLI). BLI signal intensity for each mouse was normalized the signal at day 6 following implantation (i.e. baseline) and displayed on a logarithmic scale. BLI data were combined from the same experiments used in the survival analyses (**Figure 1B**) and stratified by treatment type. Timing of BLI measurements varied slightly between experiments. Red = durable response, Blue = progressive disease.

Supplemental Figure 2. Histological and immunohistochemical profiling of GL261-luc2 and CT2A-luc tumors

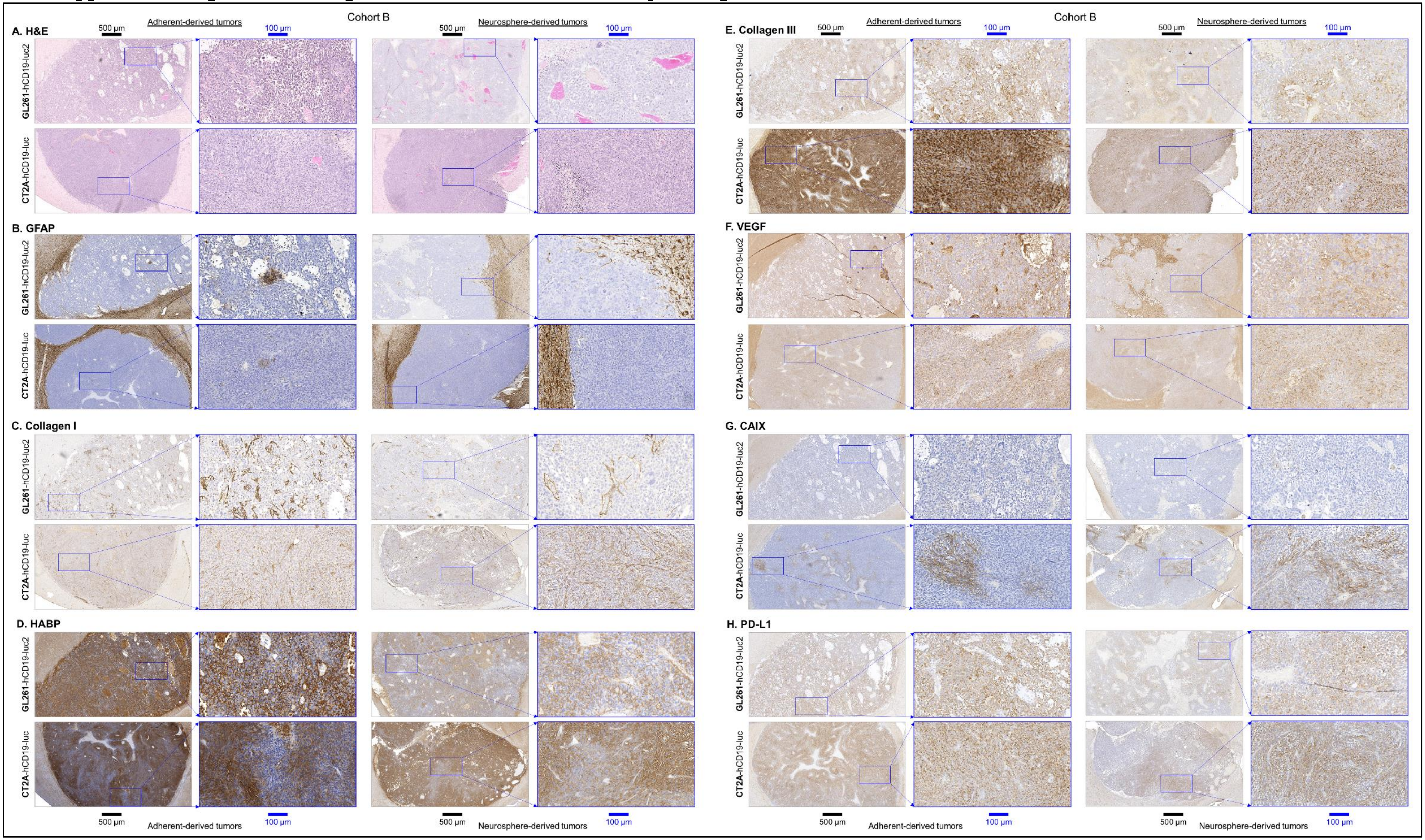

Representative **(A)** hematoxylin and eosin and immunohistochemical staining of **(B)** GFAP, **(C)** collagen I, **(D)** hyaluronan-binding protein (HABP), **(E)** collagen III, **(F)** VEGF, **(G)** CAIX, and **(H)** PD-L1 in *ex vivo* GL261-hCD19-luc2 and CT2A-hCD19-luc tumors from experimental Cohort B. One adherent-derived and one neurosphere-derived tumor are shown for each condition. Low-magnification (scale bar = 500 µm) images with corresponding high-magnification insets in blue (scale bar = 100 µm) are shown. Representative high-magnification insets are displayed in **Fig. 1F**.

Supplemental Figure 3. Differentially expressed genes between GL261-luc2 and CT2A-luc tumors

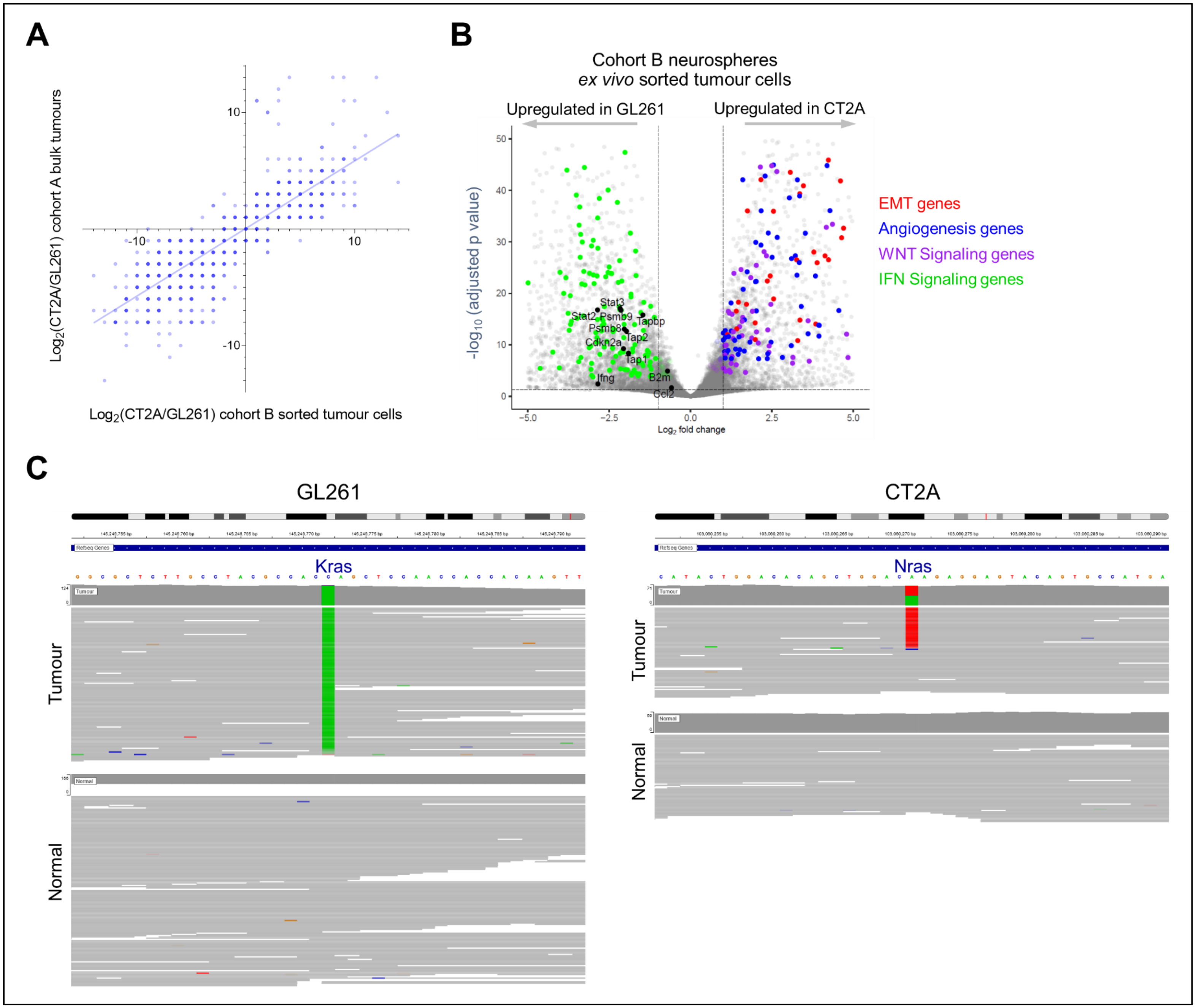

(A) For genes that were significantly differentially expressed in *ex vivo* CT2A tumors (as compared to GL261; n=3,739), the correlation of their $\log_2$-fold change between cohort A (bulk tumors) and cohort B (hCD19-sorted tumor cells). Spearman r = 0.92, p<0.001. Blue line = best-fit linear regression. Each point = 1 gene.
(B) Genes that were differentially expressed in *ex vivo* CT2A-hCD19-luc sorted tumor cells, as compared to GL261-hCD19-luc2 (n=3-5 mice each). Pre-implantation, tumor cells were cultured as neurospheres. |log2FoldChange| > 1 and a Benjamini-Hochberg FDR-adjusted p<0.05. Of 17,098 genes, 705 were beyond the plot's ranges, including *Olig2* (significantly upregulated in GL261-hCD19-luc).
(C) Whole exome sequencing of *in vitro* tumor lines and normal C57/BL6 mouse tail identified a *Kras* c.34G>T p.G12C in GL261 (*left*) and an *Nras* c.182A>T p.Q61L in CT2A (*right*), as displayed using the Integrative Genomics Viewer.

Supplemental Figure 4. Deficient antigen processing and presentation machinery expression in CT2A-luc

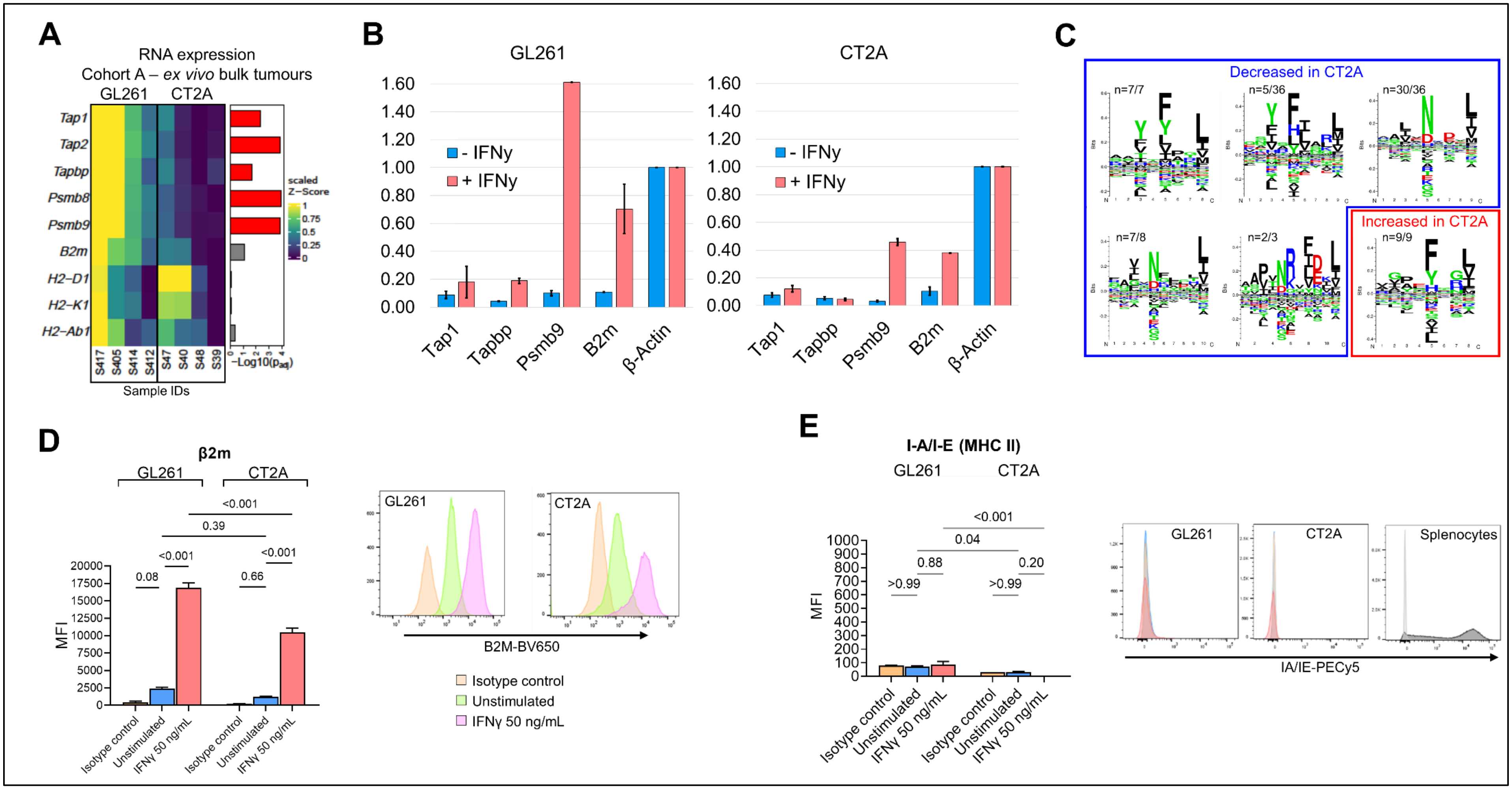

(A) Heatmap depicting the differential RNA expression of antigen processing and presentation machinery genes in *ex vivo* bulk GL261-luc2 (n=4 mice) and CT2A-luc (n=4 mice) tumors, with the corresponding FDR-adjusted p value. Expression values were row normalized, Z-scored, bounded, and scaled. Red = FDR-adjusted p value<0.05.

(B) Bar charts depicting the densitometry of western blot bands from **Figure 3D** (cropped blots that contain one of the replicates) and **Supplemental File 1** (uncropped blots that contain both replicates) for in vitro GL261-luc2 and CT2A-luc cell lines that were treated with (red) and without (blue) IFN-γ stimulation. Protein band signals were normalized to their respective B-actin bands, averaged between the two replicates, and plotted as mean +/- standard error of the mean.

(C) Among the MHC class I peptides that were differentially presented between GL261-luc2 and CT2A-luc *ex vivo* tumors (n=3 mice each), the shared amino acid motifs that were decreased (blue) or increased (red) in CT2A-luc.

(D) *Left*: β2M surface expression median fluorescence intensity (MFI) detected by flow cytometric analysis on *in vitro* GL261-luc2 and CT2A-luc cells that were either stimulated with 50 ng/mL IFN-γ or unstimulated. Isotype controls were included as a baseline. Expression was analyzed using one-way ANOVA, with two-sided pairwise p values adjusted for multiple testing using the Holm-Šídák method. The experiment was conducted in triplicate, bars = mean ± standard error. *Right*: Representative histograms of β2M expression.
(E) *Left*: MHC class II surface expression median fluorescence intensity (MFI) detected by flow cytometric analysis on *in vitro* GL261-luc2 and CT2A-luc cells that were either stimulated with 50 ng/mL IFN-γ or unstimulated. Isotype controls were included as a baseline. Expression was analyzed using one-way ANOVA, with two-sided pairwise p values adjusted for multiple testing using the Holm-Šídák method. The experiment was conducted in triplicate, bars = mean ± standard error. *Right*: Representative histograms of MHC class II expression. Mouse splenocytes were included as a positive control for MHC class II surface expression.

Supplemental Figure 5. Differentially phosphorylated proteins between GL261-luc2 and CT2A-luc tumors

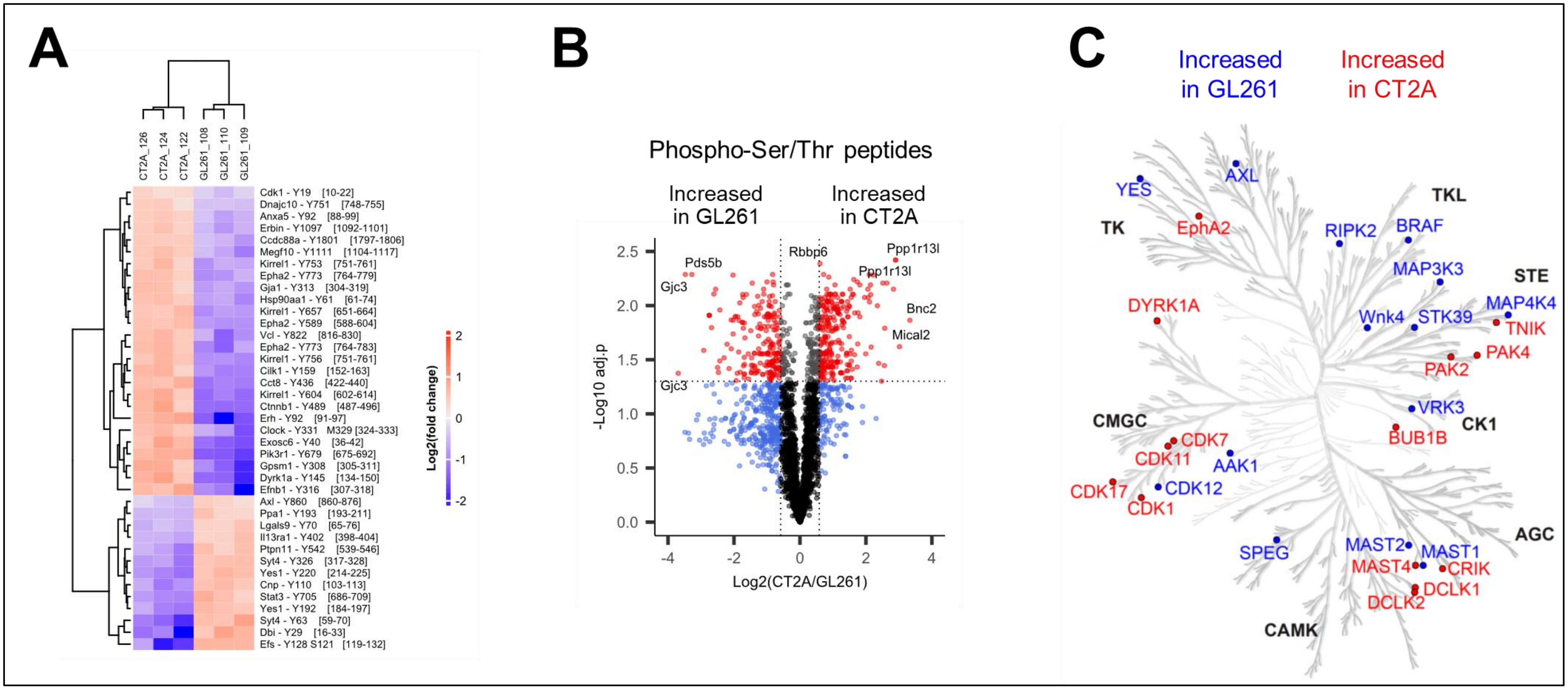

(A) Unsupervised hierarchical clustering and heatmap analysis displaying differential phosphorylation of tyrosine residues between *ex vivo* GL261-luc2 and CT2A-luc bulk tumors from Cohort A. n=3 mice each.
(B) Volcano plot displaying differential phosphorylation of serine and threonine residues between *ex vivo* GL261-luc2 and CT2A-luc bulk tumors (n=2,920 total phosphoSer/phosphoThr peptides) from Cohort A. Cutoffs included $|\log_2 \text{FoldChange}| > \log_2(1.5)$ and Benjamini-Hochberg FDR-adjusted $p<0.05$. n=3 mice each.
(C) Kinome tree displaying the significantly differentially phosphorylated kinases between GL261-luc2 (blue) and CT2A-luc (red) *ex vivo* bulk tumors from Cohort A. n=3 mice each.

Supplemental Figure 6. Secreted immunomodulatory proteins distinguish GL261-luc2 and CT2A-luc

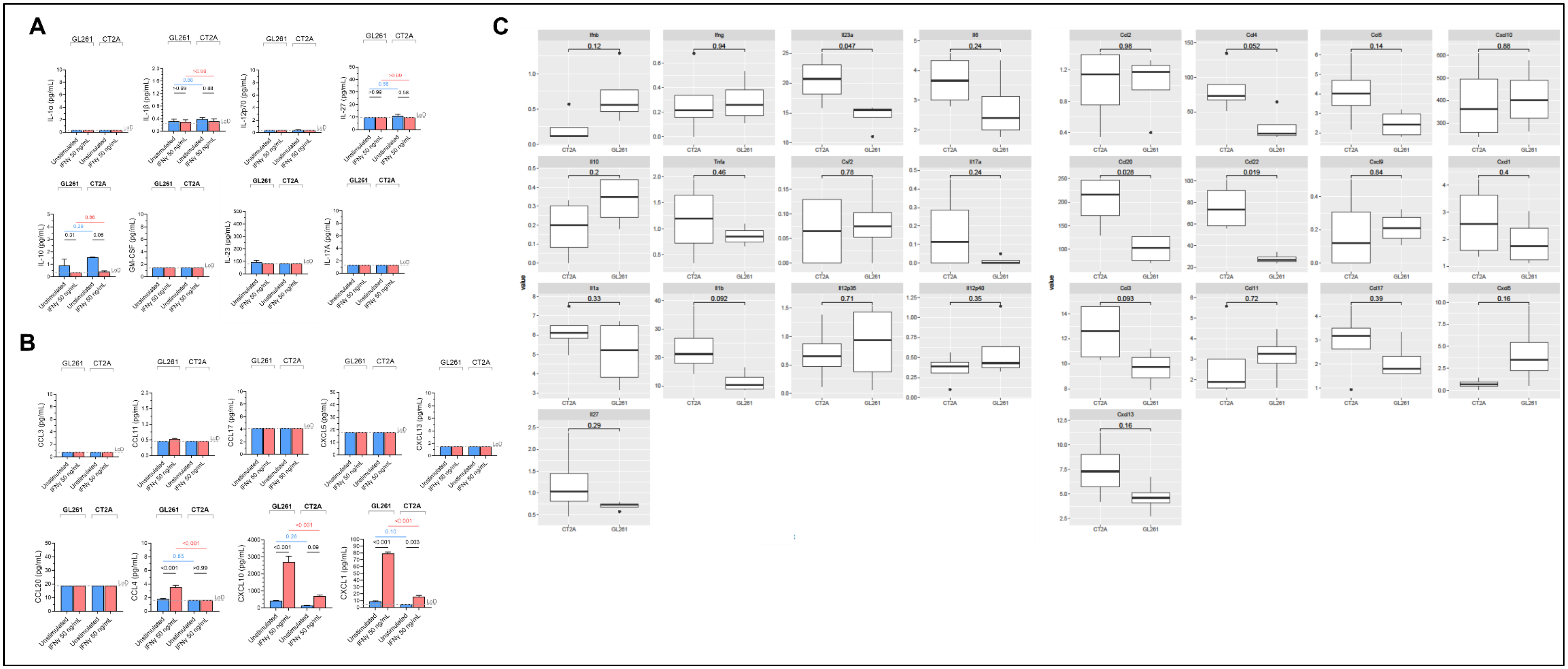

(A) Additional secreted cytokines (IL-1α, IL-1β, IL-12p70, IL-27, IL-23, IL-10, GM-CSF, and IL-17A) from the **Figure 5A** experiment that were profiled from the conditioned media of the *in vitro* GL261-luc2 and CT2A-luc cultures, which had been cultured for 24 hours without (*blue*) or with (*red*) 50 ng/mL IFN-γ.
(B) Additional secreted chemokines (CCL3, CCL11, CCL17, CXCL5, CXCL13, CCL4, CXCL10, CCL20, and CXCL1) from the **Figure 5B** experiment that were profiled from the conditioned media of the *in vitro* GL261-luc2 and CT2A-luc cultures, which had been cultured for 24 hours without (*blue*) or with (*red*) 50 ng/mL IFN-γ.

For (**A-B**), the experiment was conducted in triplicate, with secreted peptide concentrations graphed as mean ± standard error and compared using one-way ANOVA. The assay's limit of detection (LoD; *grey dashed line*) was displayed and analyzed for samples whose values were above the LoD. P values were adjusted for multiple testing using the Holm-Šídák method.
(C) Expression levels (TPM values) of cytokine and chemokine genes from the RNA sequencing data of Cohort A *ex vivo* CT2A-luc and GL261-luc2 tumors, displayed as box and whisker plots.

Supplemental Figure 7. The relationship of GL261-luc2 and CT2A-luc models to human cancer contexts

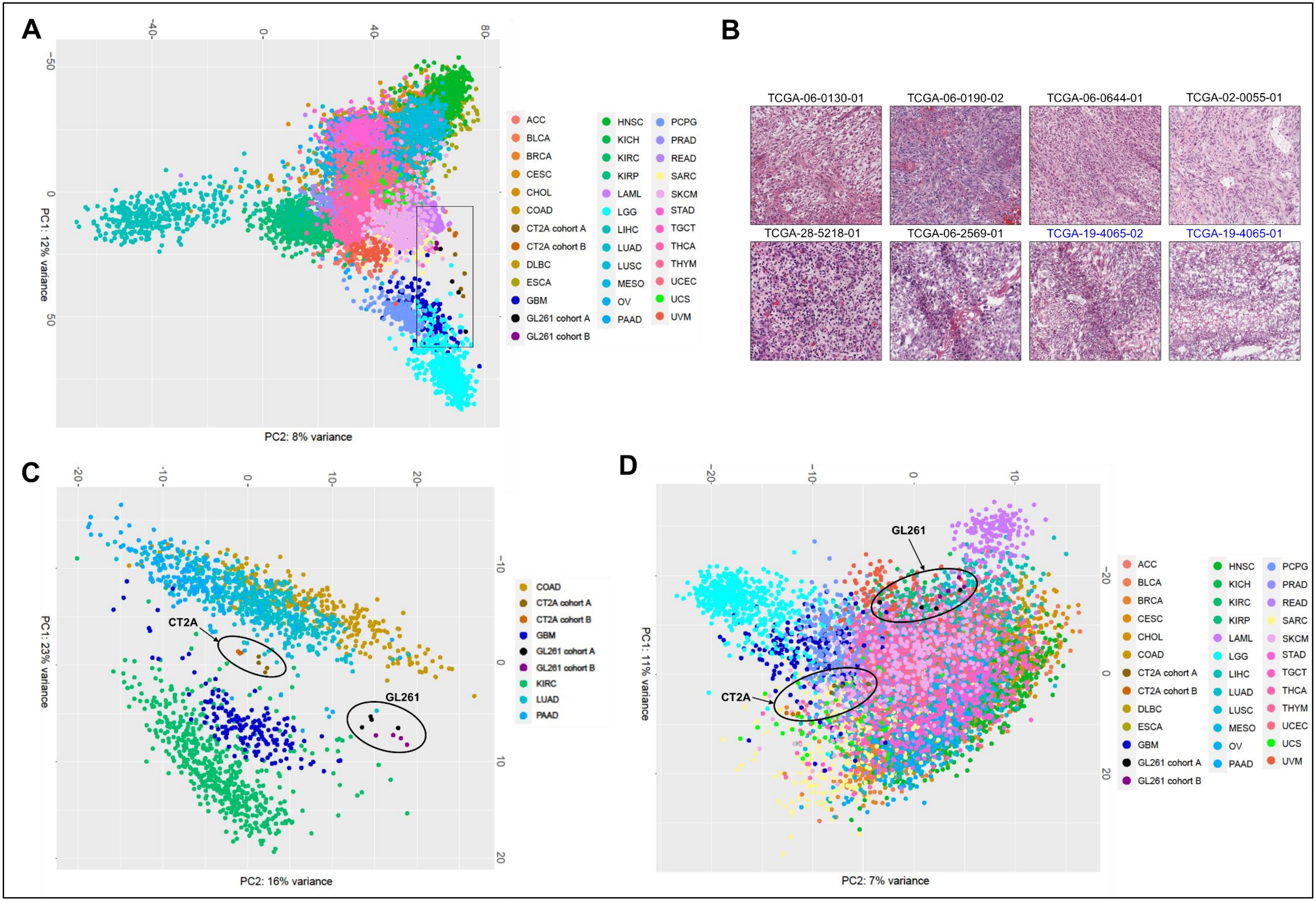

(A) Unsupervised principal component analysis of whole transcriptome expression of the *ex vivo* bulk (Cohort A) and *ex vivo* tumor sorted (Cohort B) GL261-luc2 and CT2A-luc samples alongside RNA sequencing of all human cancer samples from TCGA. Inset = higher magnification. OncoTree cancer type definitions were detailed previously.(65)
(B) Representative histological images from the 7 TCGA human glioblastoma tumor samples that closely resembled CT2A-luc tumors in unsupervised hierarchical clustering analysis of RNA sequencing data. *Blue*: the TCGA-19-4065-02 was a recurrent glioblastoma sample with mesenchymal morphology, for which the corresponding primary glioblastoma sample (TCGA-19-4065-01, which did not have mesenchymal morphology) did not cluster with CT2A-luc. Images were acquired from the Cancer Digital Slide Archive in cBioPortal.[65,66]
(C) Unsupervised principal component analysis of the gene expression between *ex vivo* GL261-luc2 and CT2A-luc tumors (circled) and human cancer samples from TCGA that commonly exhibited similar features to those that were observed in the GL261-luc2 and CT2A-luc characterizations, including RAS driver mutations (e.g., pancreatic adenocarcinoma [PAAD] and colorectal adenocarcinoma [COAD]), carcinogen-induced mutation signatures (lung adenocarcinoma [LUAD]), and mesenchymal differentiation (kidney renal cell carcinoma [KIRC]) – in addition to glioblastoma (GBM). The analysis was conducted using the member genes of the epithelial-mesenchymal transition, angiogenesis, WNT signaling, and IFN-α/γ response hallmark gene sets.
(D) Unsupervised principal component analysis of the gene expression between *ex vivo* GL261-luc2 and CT2A-luc tumors (circled) and all human cancer samples from TCGA. The analysis was conducted using the member genes of the epithelial-mesenchymal transition, angiogenesis, WNT signaling, and IFN-α/γ response hallmark gene sets.

Supplemental File. Uncropped Western Blots from Figure 3

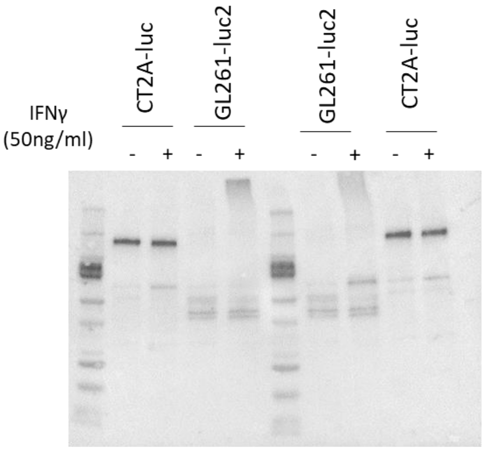

Tap1

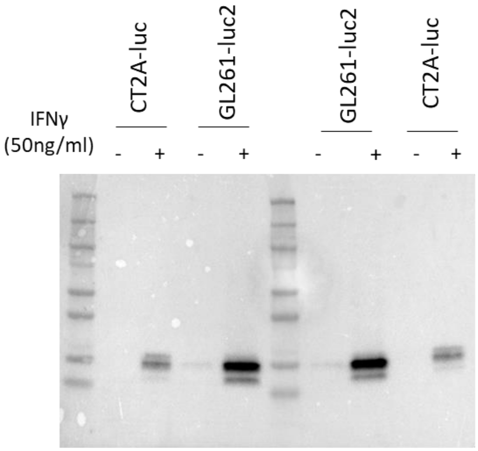

Psmb9

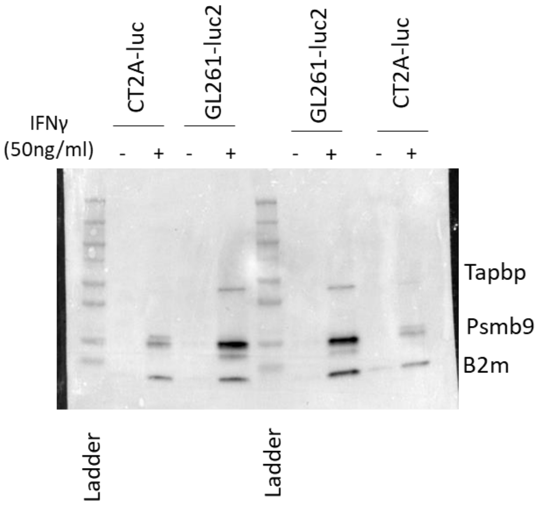

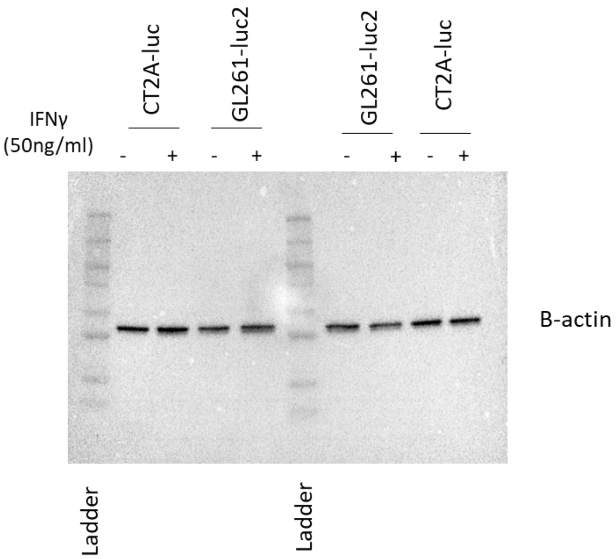

Supplement: Supplementary file 1 [file DataSheet_1.pdf]
