# Supplementary material for: Antigen presentation deficiency, mesenchymal differentiation, and resistance to immunotherapy in the murine syngeneic CT2A tumor model
Source: Front Immunol. 2023 Dec 28;14:1297932. doi: 10.3389/fimmu.2023.1297932 (PMC10782385; doi:10.3389/fimmu.2023.1297932)
Supplement: Supplementary file 3 [file Table_2.docx]

**Supplemental Antibodies Table**

| **Antibody (dilution)** | **Source** | **Identifier** |
| --- | --- | --- |
| VEGF (1:50) | Thermo Fisher Scientific | Cat#MS-1467-P |
| Collagen III (1:500) | Abcam | Cat#Ab7778 |
| PD-L1 (1:100) | Cell Signaling | Cat#13864 |
| GFAP (1:1000) | Dako | Cat#Z0334 |
| CA-IX (1:1000) | Abcam | Cat#Ab15086 |
| Collagen I (1:125) | Abcam | Cat#Ab21286 |
| HABP (7.5ug/ml) | Millipore | Cat#385911 |
| H-2Kb (1:100) | BioLegend | Cat#116507 |
| H-2Db (1:100) | BioLegend | Cat#111513 |
| I-A/I-E (1:100) | BioLegend | Cat#107611 |
| β2-Microglobulin (1:100) | BD biosciences | Cat#745291 |
| Rat IgG2b, κ Isotype Ctrl (1:100) | BioLegend | Cat#400609 |
| Mouse IgG2a, κ isotype Ctrl (1:100) | BioLegend | Cat#400212 |
| Mouse IgG2b, κ isotype Ctrl (1:100) | BioLegend | Cat#400320 |
| PD-1 | Gordon Freeman | 332.8H3 |
| IgG1 isotype control | BioXCell | MOPC21 |
| CTLA-4 | BioXCell | 9D9 |
| OX40 | BioXCell | OX-86 |
| IgG1 clone isotype control | BioXCell | HRPN |
| Tap1 (1:1000) | Proteintech | Cat#111114-1-AP |
| β-2 microglobulin (1:1000) | Thermo Fisher Scientific | Cat #701250 |
| Psmb9 (i.e., LMP2; 1:1000) | Thermo Fisher Scientific | Cat#14544-4-AP |
| Tapbp (i.e., Tapasin; 1:1000) | Cell Signaling | Cat#88644S |
| β-actin (1:5000 dilution) | Cell Signaling | Cat#5125S |
| rabbit secondary antibody | GE Healthcare | Cat#NA934 |
